# Parametric Analysis and Stiffness Investigation of Extended End-Plate Connection

**DOI:** 10.3390/ma13225133

**Published:** 2020-11-13

**Authors:** Liang Luo, Maohua Du, Jian Yuan, Jun Shi, Suhui Yu, Yi Zhang

**Affiliations:** 1 School of Civil Engineering and Transportation, South China University of Technology, Guangzhou 510641, China; ctluoliang2018@mailscut.edu.cn; 2 Ordnance Engineering College, Naval University of Engineering, Wuhan 430033, China; 3 Academy of Combat Support, Rocket Force University of Engineering, Xi’an 710025, China; yuanjian@hit.edu.cn (J.Y.); yusuhui1988@126.com (S.Y.); zhangyi_yantai@163.com (Y.Z.); 4 School of Civil Engineering, Central South University, Changsha 410075, China; csushijun@csu.edu.cn

**Keywords:** finite element analysis, initial rotational stiffness, component method, parameter analysis, semi-rigid connection, column top-side loading, extended end-plate

## Abstract

Extended end-plate (EP) bolted connections are widely used in steel structures as moment-resisting connections. Most of these connections are semi-rigid or in other words flexible. The paper aims to study the behavior of such connections under the effect of column top-side cyclic loading using the finite element (FE) method. For semi-rigid connections, it is very vital to determine the moment-rotation relationship as well as the connection stiffness. These beam-column connections have been parametrically studied, the effect of joint type, shear forces, diameter of bolt, thickness of end-plate, and end-plate style were studied. Parametric studies show that the panel zone shear force is the key factor and has a significant effect on the connection stiffness. Finally, based on the component method, the stiffness of the bending component is improved, and the initial stiffness calculation model of the connection under column top-side cyclic loadings is established. The results show that the calculation model is in good agreement with the finite element analyses, and this proves that the calculation model proposed in this study could act as a reference method.

## 1. Introduction

Both the 1994 Northridge earthquake and the 1995 Hyogoken-Nanbu earthquake [1,2,3,4,5] resulted in widespread and unanticipated failures in steel frame beam-column welded joints. Plenty of investigations and researches indicate that the beam-column connection failure was caused by the brittle fracture of welds. The seismic behavior of semi-rigid connections, which exhibit better ductility than welded connections, has been widely studied. The semi-rigidity of the beam-column joint means that the relative rotation changes when the joint is subjected to certain moment, and the joint has certain rotational stiffness. The current design codes [6,7,8] of many countries require the moment-rotation (*M-θ*) relationship curve of the joint as the design basis.

Krishnamurthy, N. et al. [9] used flexible bolt connections for the first time in the United States, who developed a 3D FE analysis model and analyzed the influence of the end-plate thickness on the bolt connections; while Shi, G. et al. [10] compared the influence of dimensional parameters on the joint performance of eight flushes and extended end-plates and analyzed that the joint rotation mainly derives from the relative deformation between the end-plate and the column flange and the shearing deformation of the panel zone, respectively. Tao, W. et al. [11] used experiments and FE methods to analyze the space frame, considering the flexural resistance performance of the plane joint connected by the weak axis of the end-plate, and focused on the deformation capacity and the lateral stiffness of the frame. Abidelah, A. et al. [12] studied end-plate connections with or without stiffeners, the experimental results are analyzed on the basis of the global moment-rotation curves and the evolution of the tension forces in the bolts. The research parameters such as rotation capacity and rotation stiffness are given. ElSabbagh, A. et al. [13] considered monotonic and cyclic loading conditions, using the FE parameter model to analyze the difference in the mechanical behaviors of the extended end-plate connection. While Grimsmo, E.L. et al. [14] studied the dynamic loading conditions and found that the ductility of the end-plate joints was improved. Chen, S. et al. [15] proposed a stiffness calculation formula suitable for spatial joints by improving the EC3 component method. Stiffness calculation difference between plane joints and spatial joints connected by the end-plate were compared. Zhan, W. and Tao, W. [16] studied the flexural behavior of the weak-axis and strong-axis end-plate connection and concluded that the two types of connections are still typical semi-rigid connections. D’Alessandro, E. et al. [17] provide the automatic tool that allows easy-to-use application of the component method [18] for the evaluation of joints stiffness and strength, and carried out parameter analysis on the two end-plate beam-column joints.

Sherbourne [19] and Gang et al. [20] developed an FE numerical model to study the mechanical properties of the beam-column end-plate connection, focusing on the moment-rotation relationship of the connection. Modeling details are given, and all bolts are pre-tightened. However, there is a lack of data to study the force transmission mechanism of each component at the end-plate connection. Guo et al. [21] compare the hysteretic behavior, stiffness, and strength of stiffened and unstiffened extended end-plate connections of beam-column joints, the stiffeners have higher bearing capacity and energy dissipation capacity and increase the stiffness of the connection, which has an important influence on its cyclic behavior. Additionally, Wang Yihuan et al. [22] did research on the cyclic behavior of a new type of anchoring blind bolt extension endplate joint, Zhao Dongzhuo et al. [23] analyzed the incomplete similarity error of the end-plate connecting the steel frame.

The moment-rotation behavior of the end-plate joint with an extended stiffener was investigated by Tartaglia et al. [24], and also, the design method was developed by Francavilla et al. [25]. Katalin and Miklós [26] investigated a modified component method that could be applied to the analysis of spatial joints. Additionally, A. Loureiro et al. [27] studied a spatial joint composed of a strong axis and a weak axis connected by an end-plate. Experiments and numerical studies were carried out to evaluate the interaction between both axes. There is a coupling effect between the two, and the initial stiffness is slightly increased. Pu Yang et al. [28] proposed a phenomenological component-based model with several separated springs, taking into account the local response of other components, to study the seismic performance of bolted end-plate connections.

Most of the above studies conform to the “strong column and weak beam” design criteria. The test, finite element, or both, simulations are carried out under the condition of beam tip loading. However, in actual engineering, for example, considering the combined effect of the floor slab and beam, the stub effect is caused by opening windows in the filling wall, and so on [29,30]. These failure phenomena show the law of “strong beams and weak columns” and a new loading method is needed to simulate the real stress state of the joints under the above-mentioned failure modes. The column top-side loading method is undoubtedly an acceptable choice. Compared with the beam tip and the column top-side loading, there are differences in the rotation angle measurement method, the force transmission mechanism, the failure model, and the semi-rigidity of the joints, which urgently need to be quantitatively studied.

To further study the mechanical behaviors of the extended end-plate under the column top-side loading, this paper includes three main phases. The first phase is based on our previous experimental research [31] using the finite element analysis (FEA) for verification, which was based on the previous three intermediate column joints (IC-EP1/2/3) and side column joints (EC-EP1/2/3). While the second phase focuses on the parameter study of the 144 (3D) finite element model by changing the geometric dimensions for effective parameters, such as column section, beam length, end-plate thickness, and bolt diameter to determine the influence of these parameters on semi-rigid characteristics such as bending resistance and initial rotational stiffness values. In the third phase, the calculation formula of component stiffness was improved, and a calculation model of joint initial rotation stiffness based on joint deformation was proposed, and verified with test data and parametric FE model.

## 2. Test Overview

**Figure 1:**
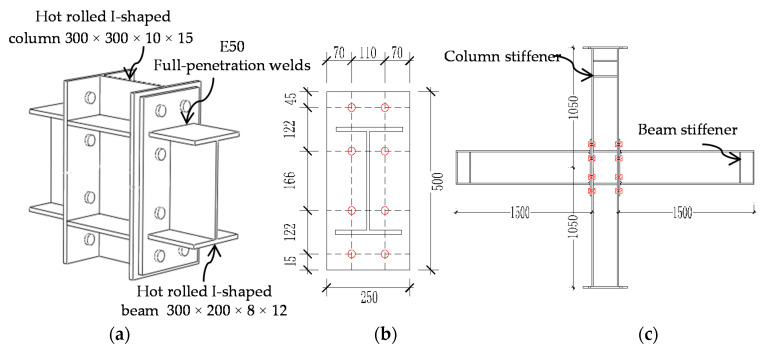
Details of joint specimens (dimensions in mm): (**a**) Detail drawing of the connection; (**b**) layout of the end-plate and bolts; (**c**) overall size of intermediate column (IC) joints [31].

**Figure 2:**
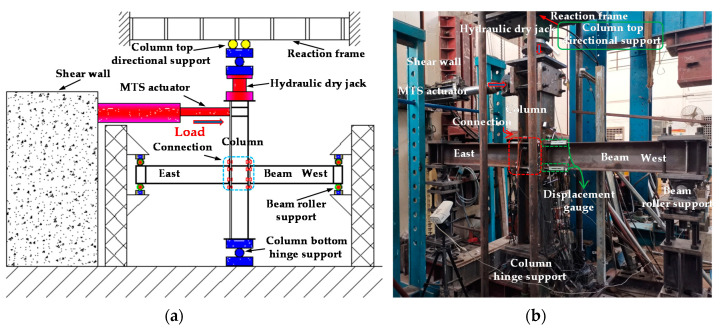
Test setup: (**a**) Schematic of test setup and (**b**) laboratory test setup [31].

In the experiment, six joint specimens of extended end-plate connections were designed, namely, the intermediate column joints (IC-EP1/2/3) and the edge column joint (EC-EP1/2/3), respectively. These joints were designed according to the standard for the design of steel structure [32], and the specimen size was derived from an actual high-rise building project. The length of the beam and the height of the column depend on the position of the frame anti-bending point. The details of the specimens are shown in Table 1. The variable parameters were the joint type, end-plate thickness, and bolt diameter. The beam was connected to the column by 10.9-grade friction high-strength bolts. Construction of high-strength bolts adopted the torque method to tighten. The initial tightening torque and final tightening torque of 10.9-grade M20 and M24 high-strength bolts are 280–446 N·m and 400–760 N·m, respectively. The corresponding pre-tightening (*F_pre_*) of the two kinds of bolts is 155 kN and 225 kN. The beams and columns of the six test specimens all adopt hot-rolled I-shaped sections, the steel strength grade of all components of the joint is Q345B, and the basic configuration of the joints are shown in Figure 1. The end-plates and beams are connected by fully-penetrating butt welds, and all welds in the test specimen are first-grade welds. The friction surface of the connected member was prepared by sandblasting to obtain a friction coefficient of 0.44. The above two processes are completed in the factory standard workshop. A 1250 kN axial load was applied to the column top and remained constant by using a hydraulic jack. The ratio of the axial load was 0.3 for the columns. *Fc* = 0.3 × *fc* × *Ac* = 1250 kN, where *fc* takes the nominal yield strength of Q345B steel 345 Mpa, and *Ac* is the column section area. The test data of the material properties of the joint components and the bolts have been used as the parameter values of the FE material model in Section 3.1. For the intermediate column (IC) joint connections on both sides, and they were labeled as the west connection and east connection, as shown in Figure 2.

**Figure 3:**
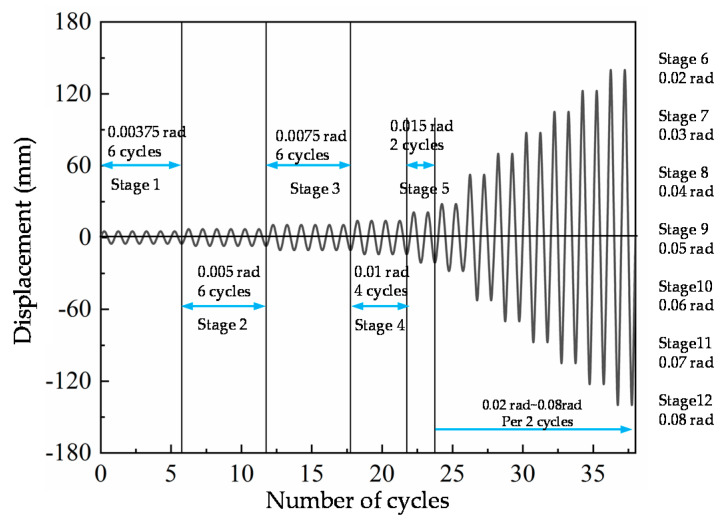
Loading protocol.

The test setup is shown in Figure 2. The column was connected to the MTS hydraulic actuator through the loading plate. The beam tips are roller supported, the column bottom is hinged to the foundation, and the column top is connected to the reaction frame through directional support. Refer to the loading protocol of SAC joint venture (1997) (Figure 3), and the loading was controlled by story drift displacement in the whole process. The test terminated when joint failure occurred or the load device limit was reached.

**Figure 4:**
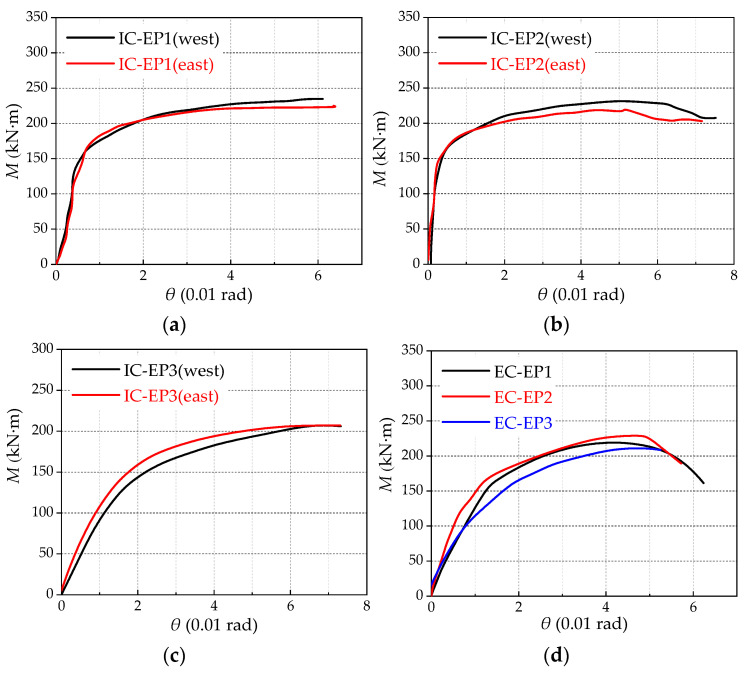
Moment-rotation skeleton curves for specimens: (**a**) IC-EPl specimen; (**b**) IC-EP2 specimen; (**c**) IC-EP3 specimen; and (**d**) EC-EP1/2/3 specimen.

The moment-rotation responses for all specimens are illustrated in Figure 4, and the test results are provided in Table 2. (The specimens IC-EP1/2 and EC-EP1/2 refer to previous research work [31]), and the specimen IC-EP3/EC-EP3 are newly added test data. Figure 4a,b shows that slight differences existed between the west connection and east connection. This conforms to the laws of mechanics, and both side connections with identical parameters are the same mechanical behaviors under symmetrical loading. However, compared with Figure 4c,d the initial rotational stiffness is quite different. This is because of the two forms of joint panel zones in different stress states and boundary conditions.

## 3. Finite Element Modeling

The extended end-plate connection analysis model was established using Abaqus Standard^®^ [33] module. The nonlinear finite element (FE) method can save expensive cost and time of experimental work, and effectively avoid uncontrollable errors during test processes, which can intuitively reflect the stress distribution of each component in the FE simulations. On one side or both sides of column flanges, set extend end-plate connected to the beam as the object of this study, that is, intermediate column (IC) joint and edge column (EC) joint. The type and loading method of these research joints were evaluated. The symmetrical boundary conditions were worth considering, hence, the half-model (FE 1/2) and the quarter-model (FE 1/4) were established. These numerical models can reduce the occupation of storage space; meanwhile, an exploratory finite element analysis was conducted for the subsequent parameter analysis, expecting to find a model with moderate mesh density and acceptable accuracy in results. Additionally, the FE model that has been developed can effectively verify the six joint test data, paving the way for the content of the following section.

### 3.1. Material Models

**Figure 5:**
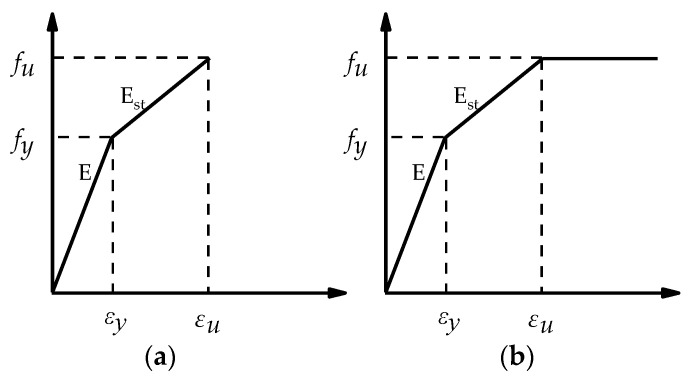
Material constitutive model: (**a**) Bolt constitutive model and (**b**) steel constitutive model [31].

The stress-strain relationship of the steel can be the simplified trilinear model and considers the plastic hardening of the material. The Von Mises yield criterion is adopted to determine whether the steel reaches the yield point in the multi-axial stress state. When the equivalent stress of the steel exceeds the yield stress, the steel will undergo plastic deformation. A bilinear kinematic hardening model was applied to the high-strength bolt constitutive, which is very suitable for high-strength steel. The material parameters of the FE model correspond to every actual tensile coupon test result to better verify the mechanical properties of the joint. Poisson’s ratio was assumed to be 0.3. See Table 3 for detailed information about the material properties of steel and bolts. The stress-strain relationship of the steel and bolt can be simplified as Figure 5 with the material constitutive curve presented by Bahaari, M. and Sherbourne, A.N. [34].

### 3.2. Finite Element Modeling

**Figure 6:**
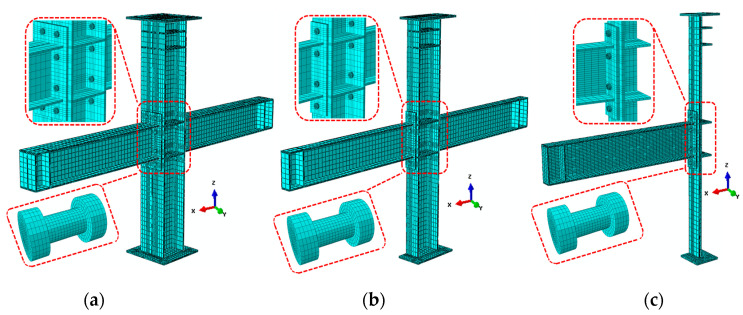
IC joint model and mesh of steel part: (**a**) Full-model (FE all); (**b**) half-model (FE 1/2); and (**c**) quarter-model (FE 1/4).

**Figure 7:**
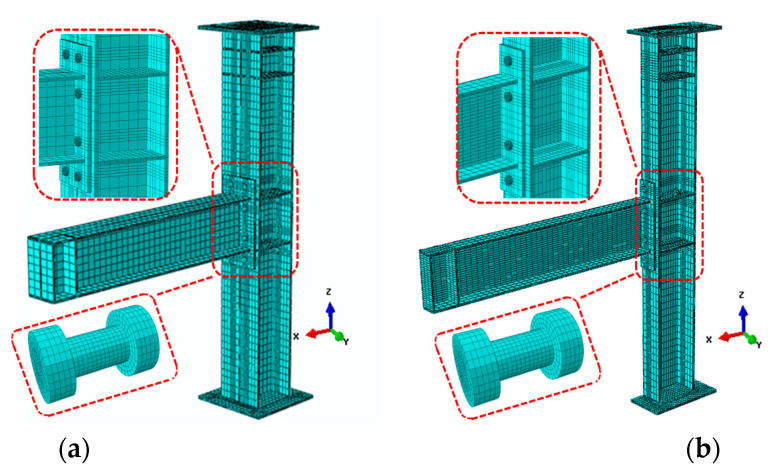
EC joint model and mesh of steel part: (**a**) Full-model (FE all) and (**b**) half-model (FE 1/2).

All parts were modeled using the 8-node linear brick incompatible mode element (C3D8R), which reduced integration and used hourglass control. The model is divided into five parts, namely, beams, columns, end-plates, bolts, and web stiffeners. Tie contact was used for the welding relationship between the end-plates and steel beam and does not consider other weld modeling. The general mesh size for the entire model was moderate mesh density, and there were at least three layers in the thickness direction. All components were controlled by a structured mesh. The above-mentioned IC joint of the full model (FE all), the half-model (FE 1/2), and the quarter-model (FE 1/4) corresponding the total number of elements is about 104,000, 52,000, and 26,000 elements, respectively, and normal hard contact to simulate the extrusion phenomenon between bolts and plate. Tangential penalty function was used to simulate friction between the end-plate and the column flange (the friction coefficient of 0.44). The shapes and mesh division diagrams of the components are shown in Figure 6 and Figure 7.

### 3.3. Boundary Conditions and Symmetry

Considering the symmetry boundary conditions and the loading direction, to divide an appropriate element mesh, the accuracy of the finite element analysis result can be guaranteed. Due to the difference of boundary conditions, the IC joint adopts half-model (FE 1/2) and quarter-model (FE 1/4), and the EC joint only adopts half-model (FE 1/2) for simulation calculation. The above symmetrical models combined themselves into a full-model (FE all), for specific values refer to Table 4, which were compared with the experimental results and aim to explore a kind of analysis model. The model not only meets the requirements of analysis accuracy but also saves calculation costs as much as possible, and does an exploratory study for the parameterized analysis in the subsequent sections. The boundary conditions of the model are consistent with the experimental settings of the specimen, the symmetrical model only deforms in the XOZ plane, so the initial setting limits the UY translation direction and RX and RZ rotation. The top of the column is directional support, which corresponds to a restricted rotation in the RY direction; the bottom of the column is hinged support, this restricts UX and UZ translation; and the beam tip is roller support, which only restricts UZ translation.

### 3.4. Loading Type

In order to simulate the loading effect and boundary constraint of the beam tip and the column top-side, coupling constraints are applied to the specified area to eliminate unrealistic stress and strain concentration. Each model applies three types of loads, the first was the constant load that was applied at the middle of the bolt shank to simulate the bolt pretension force, the second is to maintain an axial compression ratio of 0.3 and apply a constant pressure value on the column-top, while the third was the cyclic load that the displacement was applied in the form of small steps at the column top-side to generate the moment on the connection. The cyclic displacement history of FE is similar to the experimental loading protocol for increasing amplitude, and both were incrementally imposed.

## 4. Finite Element Analysis and Test Result Comparison

**Figure 8:**
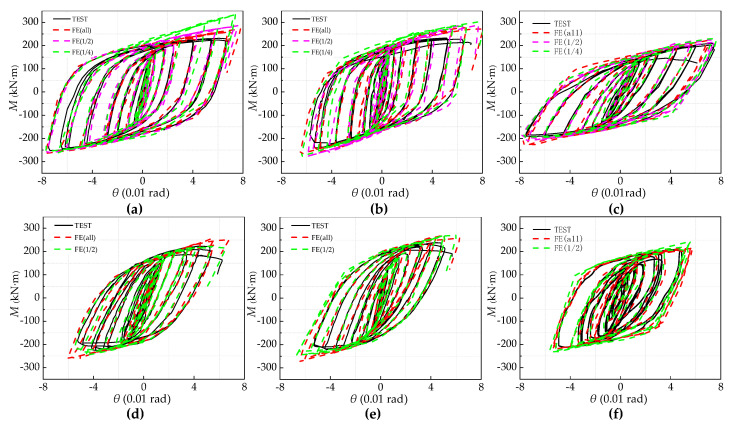
Comparison between experimental and FE results for the four tested specimens. *M-θ* skeleton curves: (**a**) IC-EP1/west; (**b**) IC-EP2west; (**c**) IC-EP3west; (**d**) EC-EP1; (**e**) EC-EP2; and (**f**) EC-EP3.

These FE models were established and solved by using Abaqus software (Version 6.14, SIMULIA, RI, USA). As shown in Figure 8, the FE and test hysteresis curves for all specimens show satisfactory agreement. As the load-displacement increases in the later stage, local differences begin to appear between the two. This is because the constitutive relationship of the steel used for the FE is that the elastoplasticity of kinematic hardening is different from the constitutive curve of the actual material in the test, and the initial geometric defects and testing errors make FE and testing slightly different. However, the error range is within the controllable area, and the finite element analysis and test hysteresis curves of all models show satisfactory consistency.

**Figure 9:**
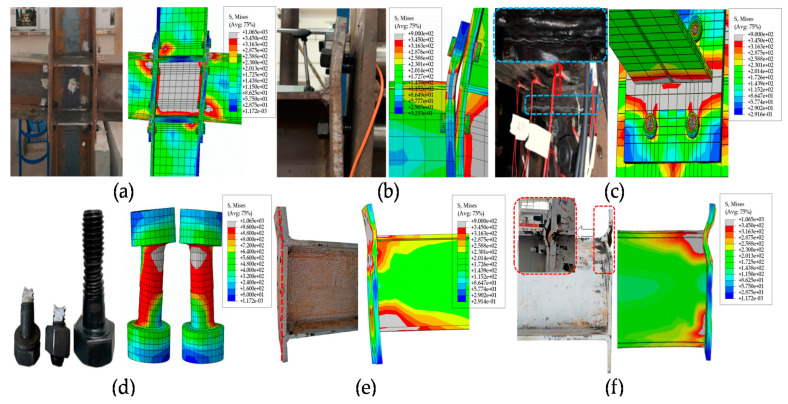
Comparison of test and finite element: (**a**) Panel zone shear deformation; (**b**) excessive bending of the end-plate at the beam upper flange; (**c**) specimen IC-EP1 connection weld cracking; (**d**) bolt break; (**e**) specimen IC-EP3 thick end plate straight bending; and (**f**) specimen EC-EP3 thin end plate curved bending.

As presented in Figure 9c,d, the IC-EP1 model shows that the failure mode was the welding between the beam flange and end-plate reached the ultimate stress, and then reaches the bolt yield stress, which also corresponds to the bolt breaking phenomenon in the test (in Table 2). The IC-EP2 model had serious local buckling in the column web (Figure 9a), and the failure mode was excessive shear deformation of the panel zone in the test (in Table 2). Both the numerical model and the experimental failure phenomenon of the EC-EP1/2/3 joint show large bending deformation of the end-plate. (in Figure 9b,e,f and Table 2).

**Figure 10:**
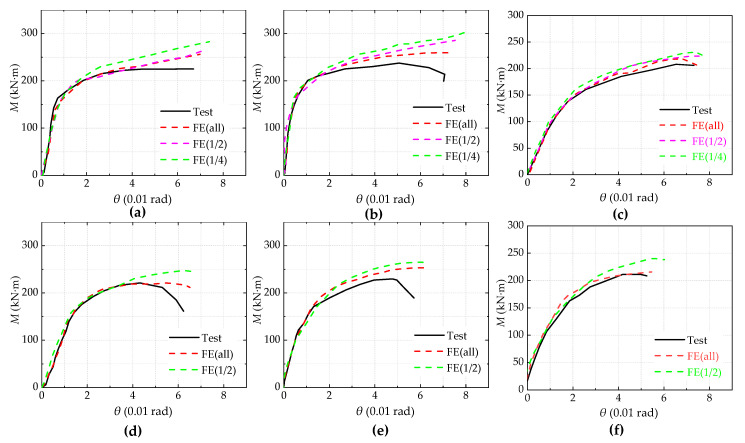
*M-θ* skeleton curves: (**a**) IC-EP1/west; (**b**) IC-EP2west; (**c**) IC-EP3west; (**d**) EC-EP1; (**e**) EC-EP2; and (**f**) EC-EP3.

As shown in Figure 10, when the joint rotation reaches 0.05 rad, it shows that all the key components of the joint have buckled, which was reflected when the joint’s ultimate moment begins to decline and the secant stiffness degrades to a very low level in the moment-rotation (*M*-*θ*) skeleton curve. The *M*-*θ* skeleton curves attained by numerical calculations in comparison with the moment-rotation skeleton curves obtained from the tests. The results show that there was good agreement between the test results and the FE simulation in the elastic stage. Additionally, in the stage after yielding, there was some disparity due to the simplification of the material constitutive behavior of the materials and test errors.

Table 5 lists the comparison between the finite element analysis and the test results. The ratio of initial rotational stiffness was defined as the finite element data divided by the test result. The mean and standard deviation of this ratio are 1.003 and 0.064, respectively. Therefore, it can be concluded that in terms of accuracy, calculation time, and storage space, the finite element analysis of the IC joint and the EC joint using the quarter-model (FE 1/4) and the half-model (FE 1/2) can meet subsequent parameter research and stiffness analysis requirements.

## 5. Parametric Research

Based on the above finite element verification and experimental research, a parameter study was conducted to develop three-dimensional FE models with variable parameters to simulate the IC joints and EC joints of the extended end-plates connection. Both material and geometry nonlinearities were considered in the analysis. This study aims to understand the connection behavior of such joints under cyclic loading on the column top-side and to determine the valid parameters on ultimate moment, rotation capacity and initial rotational stiffness.

### 5.1. Model Description

**Figure 11:**
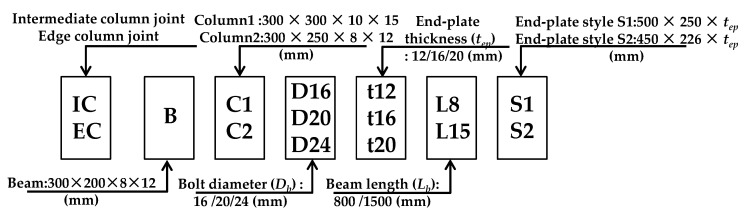
Finite element model label.

**Figure 12:**
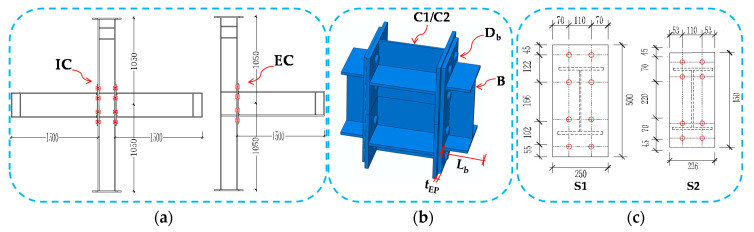
Finite element model description (dimensions in mm): (**a**) Overall size of EC joint and IC joint; (**b**) joint parameter variable; and (**c**) end-plate size.

A total number of 144 3D-FE models were created for the connection of the extended end-plate beam to column joints to study their behavior under cyclic loading. These models and the investigated parameters are summarized in Figure 11. The model contains IC joints and EC joints with the same size information, and the number of models each accounts for half; The beam of FE model uses the same section (H300 × 200 × 8 × 12 mm), The specific parameters: two groups of column profile (H300 × 300 × 10 × 15 mm and H300 × 250 × 8 × 12 mm), the beam length (800, 1500 mm), the thickness of the end-plates (12, 16 and 20 mm), the diameter of the bolts (16, 20 and 24 mm) and the end-plate styles S1 and S2, Refer to Figure 12 for specific configuration information. The type of these bolts is 10.9 high-strength bolts, and the corresponding pretensions are 58 kN, 155 kN and 225 kN, respectively. The model gives joint connection size information according to the label to create different models, and the model label number can be used to extract data for each model. Because the half mode (FE 1/2) and quarter mode (FE 1/4) of the EC joints and the IC joints are given in the above section of the FE verification, the accuracy can meet the analysis accuracy requirements. Hence, in accordance with the above-mentioned rules, the symmetric model was adopted for parametric analysis. In Table 6, model labels 001–072 are the IC joints, and model labels 073–144 are the EC joints, except for setting the difference between the single side and double side connection, both account for half and other parameters are the same.

**Figure 13:**
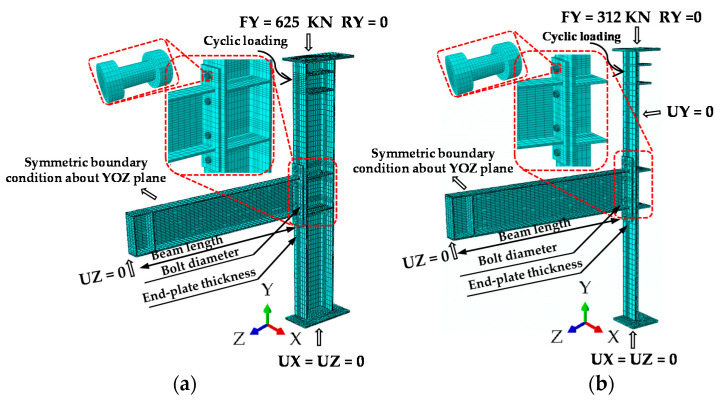
Finite element model mesh and boundary condition: (**a**) FE-1/2 model of EC joint and (**b**) FE-1/4 model of IC joint.

As shown in Figure 13, the detailed mesh and boundary conditions are given. The material model, element type, boundary conditions, and load type are consistent with the previous modeling content of the finite element verification section. The model calculation results are summarized in Table 6.

### 5.2. Results and Discussion

#### 5.2.1. Failure Modes

**Figure 14:**
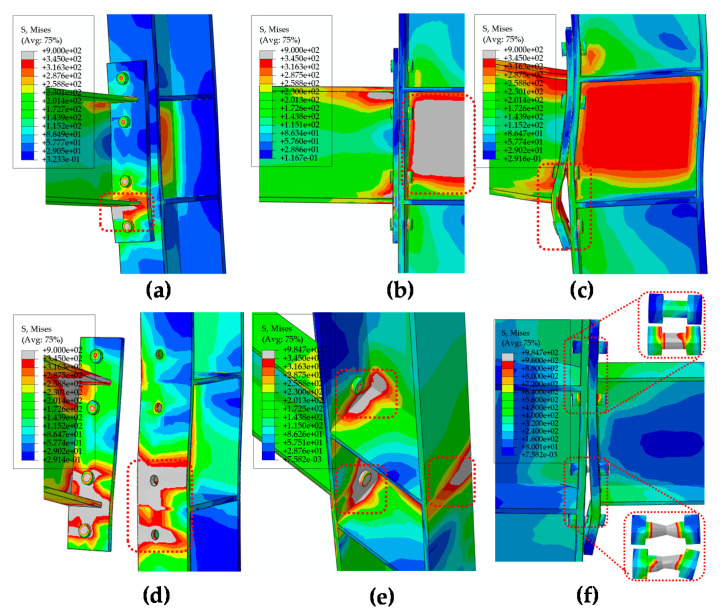
The typical failure mode of connection. (**a**) 089-ECBC1D20t16L15S1 (WEP-BF); (**b**) 023-ICBC1D20t20L15S1 (CPZB); (**c**) 091-ECBC1D20t16L15S1 (EPF); (**d**) 095-ECBC1D20t20L15S1 (CFF); (**e**) 077-ECBC1D16t16L8S1 (WCF-CS); and (**f**) 011-ICBC1D16t20L15S1 (BF).

**Figure 15:**
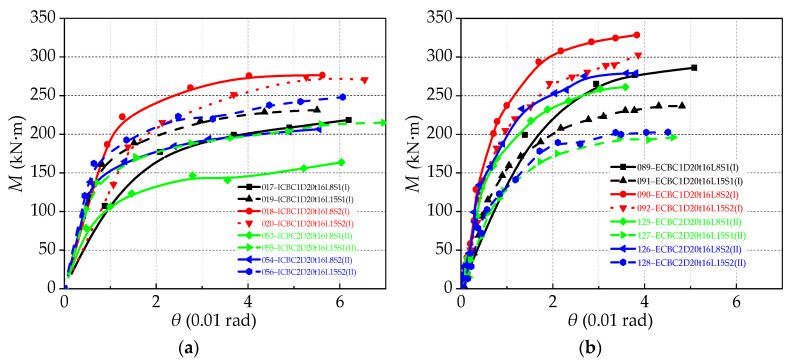
Effect of shear force (beam length): (**a**) IC joint and (**b**) EC joint.

**Figure 16:**
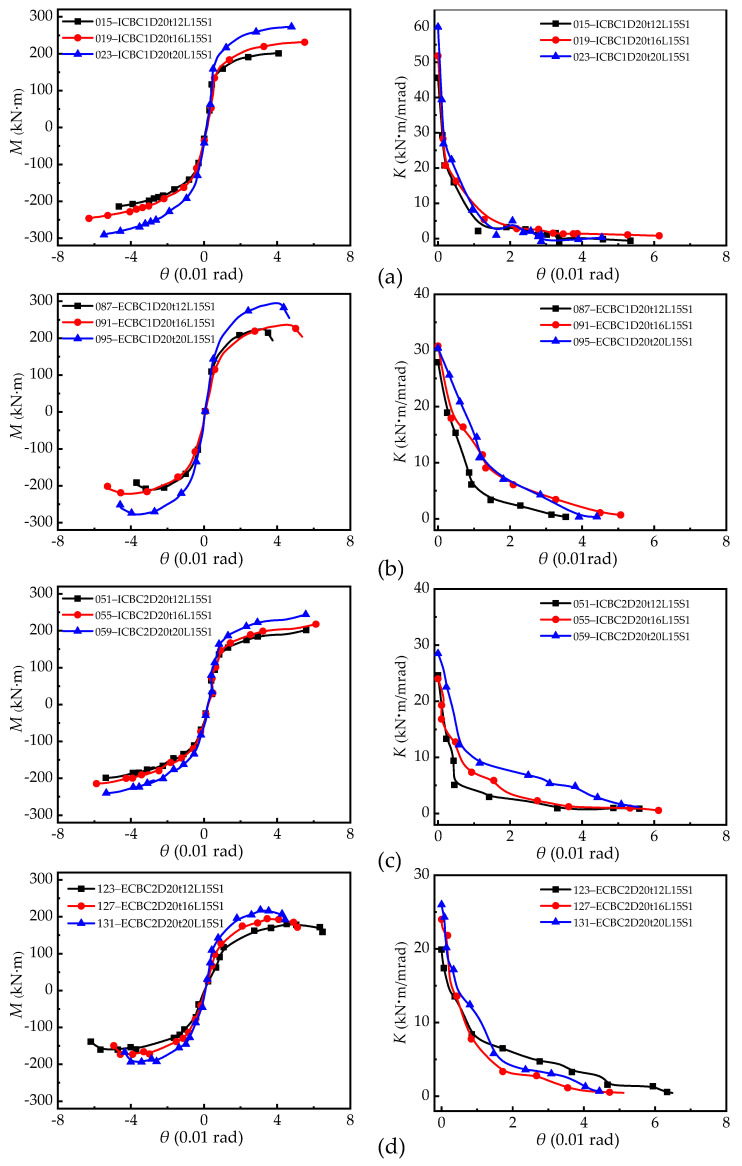
Effect of end-plate thickness on skeleton curve and stiffness degradation: (**a**) Large profile column (H300 × 300 × 10 × 15 mm) IC joint; (**b**) large profile column (H300 × 300 × 10 × 15 mm) EC joint; (**c**) small profile column (H300 × 250 × 8 × 12 mm) IC joint; and (**d**) small profile column (H300 × 250 × 8 × 12 mm) EC joint.

The FE analysis shows the different typical failure modes in Figure 14. According to the location of the failure, it can be divided into six representative failure modes, namely bolt failure(BF), column flange failure(CFF), column panel zone buckling(CPZB), end-plate failure(EPF), weld between column flange and column stiffener(WCF-CS), and weld between end-plate and beam flange(WEP-BF). As shown in Figure 14a, for connections with medium thickness end-plate and bolt diameters (89-ECBC1D20t16L8S1), the failure only occurs in the weld between the end-plates and the beam flange (WEP-BF) when it reached the ultimate stress. It can be observed that as shown in Figure 14b,c, the connections with large bolt diameter (31-ICBC1D24t16L15S1), their failure model is panel zone buckling occurs at the column web (CPZB), because of the IC joint bear enormous moment, this results in immense shear stress on the column web. By referring to Table 6, in the model (91-ECBC1D20t16L15S1), due to the EC joint only on one side connection, the deformation of the column web area is slight, yet the connection deformation is obvious. Thus the failure occurs in the end-plate (EPF). Simultaneously, for models with all thin end-plates (12 mm), the failure is almost due to excessive bending deformation of the end-plates (EPF). For the thick end-plate and crude bolt diameter models (095-ECBC1D20t20L15S1), the failure occurs at the column flange (CFF), because the connection part is strong and the column flange is relatively weak. For larger column profiles (H300 × 300 × 10 × 15 mm), the stiffener weld (WCF-CS) between the column web and the column flange has failed (77-ECBC1D16t16L8S1). In the model (11-ICBC1D16t20L15S1), the bolt reaches its ultimate stress and fails (BF). As shown in Figure 15 and Figure 16, the ultimate moment (*M_max_*) of the joint is always less than the plastic flexural resistance (*M_bp_*) of the beam or column. Therefore, the plastic hinge did not occur in the beam section, and the column section is not buckled. Failure always occurs in the connection component or panel zone.

#### 5.2.2. Effect of Shear and Column Size

The column top-side loading method is extremely sensitive to the shear effect of the panel zone, and the shear deformation of IC joints is more obvious than that of EC joints. Adopting different beam lengths to change the moments of the connections affects the shear force in the panel zone. Figure 15a,b shows two groups of curves: group (I) represents the results of the large column section (H300 × 300 × 10 × 15 mm) and group (II) represents the results of the small column section (H300 × 250 × 8 × 12 mm). In each group, the following relationships are shown: (1) beam length variation, (2) different end-plate styles, and (3) column section. For large column sections (num 017, 019, 018, and 020 in Figure 15a), the higher the shear value, the larger the shear angular rotation, and the larger the connection stiffness as well. On the other hand, for small column sections (125, 127, 126, and 128 in Figure 15b), for a lower shear force, the ultimate flexural resistance capacity is increased by 20% as a whole, but the relative rotation of the limit is reduced. Figure 15 shows that at the same value of rotation of the large column section or the small column section, the connection limit rotation is almost the same, but in considering the stiffness degradation, the IC joint with more considerable shear value has the highest stiffness at the connection hardening starting point, then the connection stiffness at the ultimate shear rotation has the minimum value (Figure 16).

#### 5.2.3. Effect of End-Plate Thickness

The effect of end-plate thickness on the moment-rotation curve of the connection is shown in Figure 16, which indicates that the connection flexural resistance capacity is increased with an increase in the thickness of end-plates, but the ultimate rotation of the connection is decreased with an increase in the thickness of end-plates, resulting in low connection ductility. Furthermore, the initial stiffness of the connection increases with an increase in the end-plate thickness. These figures show that, in most cases, increasing the thickness of end-plate causes the increase in the stiffness of the joint to vary between 4% and 15%, while the thick end-plate has the effect of slowing the degradation of the stiffness of joints. Additionally, the joints with a medium end-plate thickness (16 mm) have the most significant rotation capability. However, for the thin end-plate thickness (12 mm), its failure mode is EPF or WEP-BF. This is because the end-plate is so thin that the connection does not exert the comprehensive performance of the joint and fails prematurely.

**Figure 17:**
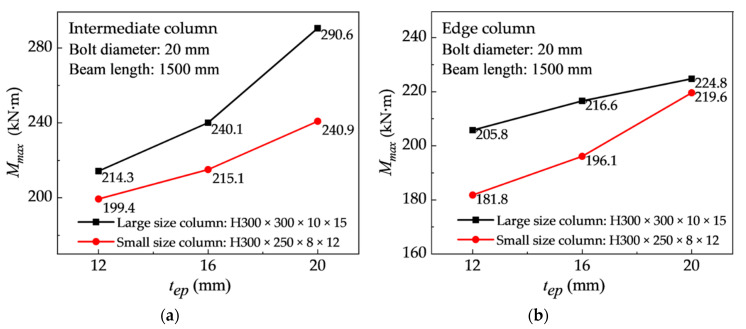
Effect of end-plate thickness on the ultimate flexural resistance of the connection: (**a**) IC joints and (**b**) EC joints.

Figure 17 shows the effect of changing the end-plate thickness on the ultimate flexural resistance of the connection. Under the condition of the same bolt diameter and beam length, the column section (large column: H300 × 300 × 10 × 15 mm; small column: H300 × 250 × 8 × 12 mm), IC joint, and EC joint are used as variables. For the small size column (H300 × 250 × 8 × 12 mm) of the connection, when the end-plate thickness is increased from 12 mm to 20 mm, the ultimate load of the IC joints and EC joints are increased by about 20%. Meanwhile, when the end-plate with a thickness of 16 mm is used, the ultimate load of the IC joint increases by about 9.7% compared to the EC joint, because the failure of the connection occurred in the column flange. For large-sized column (H300 × 300 × 10 × 15 mm) joints, when the thickness of the end-plate is increased from 12 to 20 mm, the ultimate flexural resistance of the IC joint and EC joint increases by 19% and 15%, respectively. As displayed in Figure 16, the end-plate thickness has a significant effect on the initial joint stiffness, but it only has a partial effect on the stiffness degradation of the entire loading process. The thinner the end-plate, the faster the stiffness degradation, and vice versa.

#### 5.2.4. Effect of Bolt Diameter

**Figure 18:**
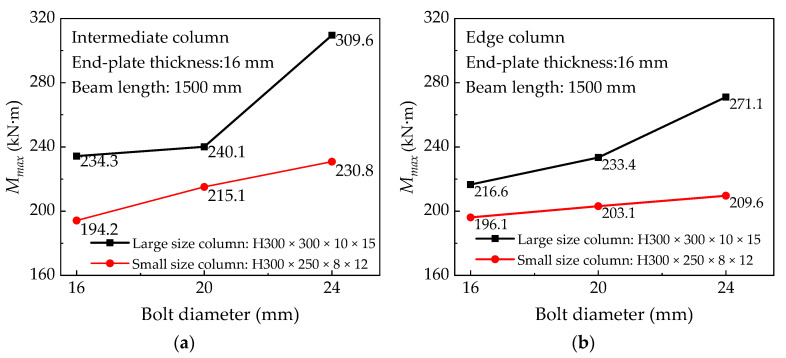
Effect of bolt diameter on the ultimate flexural resistance of the connection: (**a**) IC joints and (**b**) EC joints.

The influence of the bolt diameter on the ultimate flexural resistance of the connection is shown in Figure 18, which indicates that an increase in the bolt diameter can increase the ultimate flexural capacity of the connection, and the initial rotational rigidity will also increase (refer to Table 6). Under the conditions of the constant end-plate thickness (16 mm), beam length (1500 mm) and column section size (H300 × 300 × 10 × 15 mm, H300 × 250 × 8 × 12 mm), by changing the bolt diameter (16, 20, and 24 mm), the IC joints and EC joints are analyzed. For the larger column section group (H300 × 300 × 10 × 15 mm), regardless of whether it is an IC joint or an EC joint, when the bolt diameter increases from 16 to 24 mm, the ultimate load value increases by 32.1% and 25.6%, respectively. For the small column section group (H300 × 300 × 10 × 15 mm), the ultimate flexural resistance value only increases by about 10%. It is caused by the different weak components in the failure modes of the above two group models. On the other hand, when the bolt diameter is 20 mm, the IC joint and EC joint adopt the same column cross-section. Compared with both, the ultimate flexural resistance value of the IC joint is only slightly improved, which shows that the IC joint and EC joint are equivalent in ultimate flexural resistance capacity. By referring to Table 6, these connection joints with bolt diameter (16 mm and 20 mm) and medium thickness end-plate (16 mm and 20 mm) show excellent overall behavior of the connection in terms of the ultimate moment, joint stiffness, and ultimate rotation capacity.

#### 5.2.5. Effect of End-Plate Section Size

**Figure 19:**
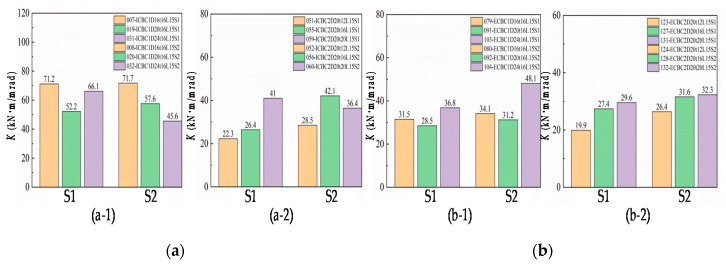
Effect of end-plate size on initial rotation stiffness: (**a**) IC joints of large and small profiles column and (**b**) EC joints of large and small profiles column.

Comparing the end-plates S1 and S2, the difference is the distance between each row of bolt holes and the end-plate size. The influence on the initial rotational stiffness of the joints is shown in Figure 19. Overall, these differences have an impact on the initial rotational stiffness of the joint. Because the bolt’s hole distance in the end-plate S2 is smaller than end-plate S1, this restricts the bending deformation of the column flange and the end-plate, making this component contribute more to the stiffness of the joint, while the ultimate flexural resistance capacity is greatly affected by the failure mode, certain weak components may fail before the end-plate.

By referring to Table 6, it is found that under the same bolt diameter, end-plate thickness, and beam length, the ultimate flexural resistance of IC joints using the S1 type end-plate connection is higher than the S2 type end-plate connection. Figure 19 shows that the initial rotational stiffness of most S2 end-plate connections is slightly greater than that of S1 end-plates and the difference between them is within the range of 1–8%. For the EC joints of small-sized column sections, the S2 end-plate form is used to improve the joint stiffness, which is better than that of large-sized column sections.

#### 5.2.6. Differences between IC Joint and EC Joint

**Figure 20:**
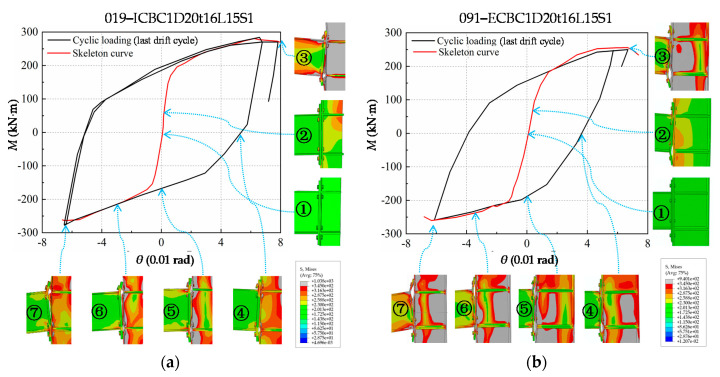
Differences between IC joint and EC joint: (**a**) IC joints and (**b**) EC joints.

Figure 20 shows the deformation of the end-plate during the cyclic loading (last drift cycle). Firstly, due to the tension in the beam lower flange, the opening between the end-plate and the column flange appears at the beam lower flange (**②,③**). Then, as the load reverses, another opening between the end-plate and the column flange begins to appear at the beam web (**④**). By further load reversal, the tension is transferred to the upper beam flange, and the opening appears between the end-plate and the beam upper flange, when the tension increased in the upper flange and compression increased in the lower flange (**⑤,⑥**), the opening between the lower beam flange and the column flange begins to close (**⑦**).

The difference between the IC joint and EC joint is whether to set up a single-sided connection or double-sided connection in the column flange, which results in the shear of the IC joint panel zone being twice the EC joint. At the ultimate loading capacity, the shear rotation of the IC joint and EC joint account for approximately 66% and 25% of the total rotation, respectively. Extracting the hysteresis curve from the cyclic load FE model shows that the characteristics of the IC joint are plumper, and their rotation span is slightly larger than the EC joint.

The size information of the 019-ICBC1D20t16L15S1 model (test specimen IC-EP1) and the 091-ECBC1D20t16L15S1 model (test specimen EC-EP1) are the same, the IC joint can enhance the joint stiffness remarkably compared to the EC joint, but the ultimate flexural resistance is basically the same. The above reason is that the strength is controlled by the failure of the weakest component. The two joint size’s information is the same, and the weakest part is also unanimous, so the strength difference is slight; while the stiffness is determined by the stiffness contribution of each component. The panel zone boundary conditions of the IC joint are significantly different from the EC joint. The component stiffness of the IC joint panel zone is much greater than that of the EC joints. Under the column top-side loading method, the stiffness of the panel zone has a significant effect on the stiffness of the entire joint, so the stiffness of the two types of joints is obviously different.

## 6. Research on the Initial Stiffness of Connection

### 6.1. Mechanical Model of the Initial Stiffness

**Figure 21:**
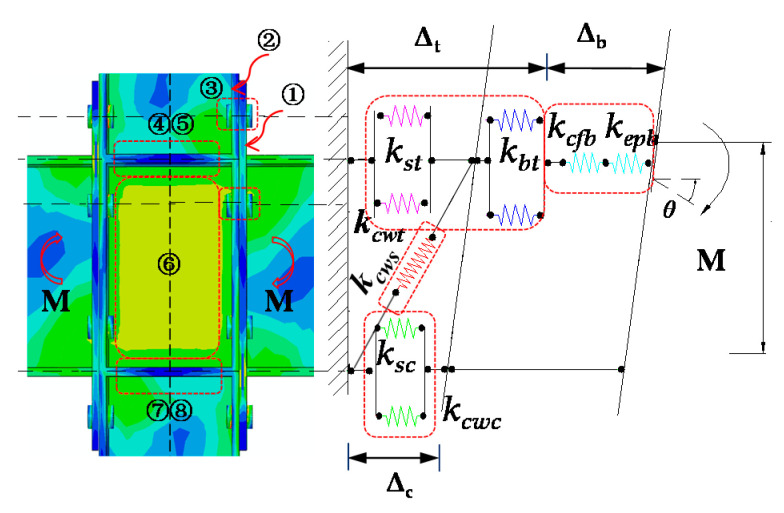
Mechanical model of connection.

The initial stiffness of the extended end-plate connection joint can be predicted according to the classical component method recommended by EC3 [7]. Meanwhile, based on the above parametric finite element research, the premise of the loading method on the top-side of the column is used to determine the components that contribute to the rotational stiffness of the end-plate connection. As exhibited in Figure 21, it can be seen that under the same directional moment, for the IC joint with end-plate connections on both sides, the shear deformation of the column web panel zone has a great influence on the initial rotational stiffness of the connection. The simplified spring model of the entire joint consists of eight springs simulating the deformation of two major parts. First, the connecting part is composed of three springs: the end-plate in bending (① *epb*), the column flange in bending (② *cfb*), and the bolt in tension (③ *bt*). Then, the column web part is composed of five springs, which simulate the shear (⑥ *cws*), tension (④ *cwt*) and compression deformation (⑦ *cwc*) of the column web, stiffener in tension (⑤ *st*) and in compression deformation (⑧ *sc*), respectively. The deformation of the entire joint is composed of these two parts, and the initial rotational stiffness of the connection can be expressed as:

(1) $$K_{ji}=\frac{1}{\frac{1}{K_{con}}+\frac{1}{K_{cw}}}$$

where *K_con_* and *K_cw_* are the stiffness of the connection and column web, respectively.

### 6.2. Calculation of Connection Stiffness

#### 6.2.1. Stiffness Calculation of the End-Plate and Column Flange in Bending

**Figure 22:**
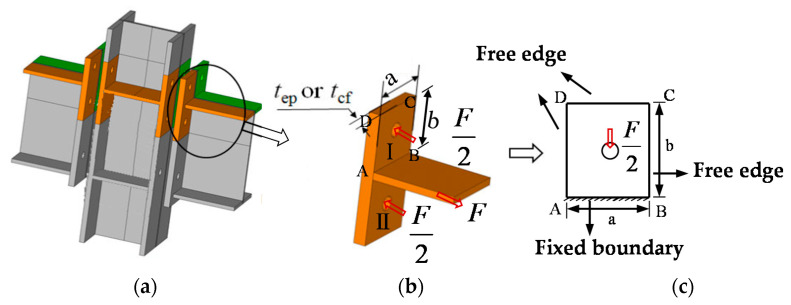
Effect of end-plate size on initial rotation stiffness: (**a**) Joint equivalent area; (**b**) Equivalent T-stub; (**c**) Cantilever rectangular plate calculation.

For the calculation of the bending stiffness of the column flange and the end-plate, the deflection at the bolt hole of the rectangular plate is used to obtain the component stiffness. As displayed in Figure 22, for the No. I plate of T-stub parts, the AB side is considered to be a fixed boundary, because the stiffness outside the plane of the beam flange is considerable, which can provide sufficient restraint for the end-plate, the remaining three sides do not provide effective constraints in terms of stiffness contribution, and can then be simplified as free sides. According to the plates and shells theory, under this boundary condition, the deflection of the rectangular plate subjected to the concentrated load *F/2* at the center can be expressed as *ω_m_* = *αFab/2D*, where *ω_m_* is the center deflection of the plate, *α* is the coefficient related to the length and constraints of the plate, *D* is the bending stiffness of the plate per unit width, *D = Et^3^/12(1 − μ^2^)*. For a rectangular cantilever plate with one fixed side and three free sides, the α coefficient reference [35] is 0.0465402. It can be concluded from the physical meaning that *ω_epb1_* = *F/2k_epb1_*, where *k_epb1_* = *1/δ_epb1_* = *D/(αab)* = *1.79* × *Et_ep_^3^/((1 − μ^2^)ab)*, *t_ep_* is the thickness of the end-plate; and the T-stub of No. II plate boundary conditions and bending stiffness are equivalent to No. I plate.

The bending stiffness of the end-plate is:

(2) $$k_{\mathrm{epb}}=\frac{3.58\times Et_{ep}^{3}}{\left(1-\mu^{2}\right)a_{ep}b_{ep}}$$

The bending stiffness of the column flange is:

(3) $$k_{\mathrm{cfb}}=\frac{3.58\times Et_{cf}^{3}}{\left(1-\mu^{2}\right)a_{cf}b_{cf}}$$

where: *E* is the elastic modulus of steel, *μ* is the Poisson’s ratio of the material; *t_cf_* and *t_ep_* are the thickness of the column flange and end-plate respectively; *a_cf_*, *a_ep_* and *b_cf_*, *b_ep_* are the calculated length and calculated height of the rectangular plate in the end-plate and column web, respectively.

#### 6.2.2. Stiffness Calculation of the Bolt in Tension

**Figure 23:**
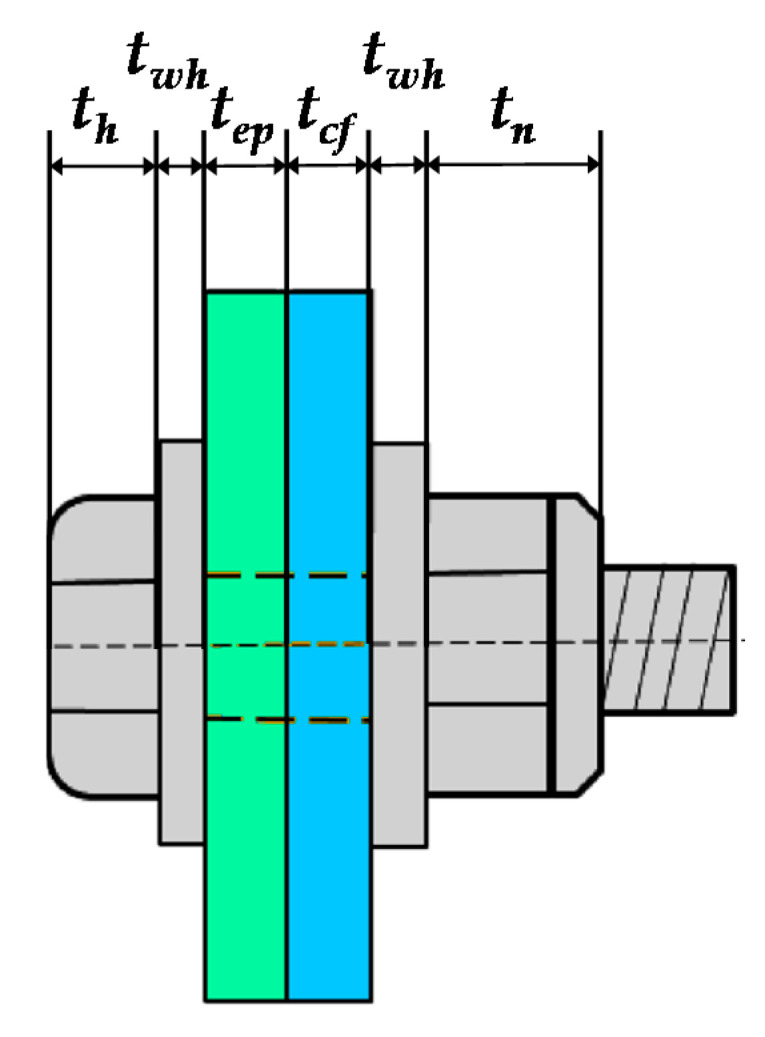
Bolt length calculation diagram.

For high-strength bolts, the pretension of the bolts significantly improves the initial stiffness of the connection. The coefficient γ is introduced in consideration of the effect of its pretension. Its value is referred to in [36], and generally γ = 10. The calculation formula of the tensile stiffness of two bolts in a single row is:

(4) $$k_{\mathrm{bt}}=2\times (1+\gamma )\frac{EA_{s}}{L_{b}}$$

where *A_s_* is the effective area of the bolt, generally 80% of the nominal area of the bolt shank; *L_b_* is the calculated length of the bolt, *L_b_* = *t_ep_ + t_cf_* + 2*t_wh_ + (t_h_* + *t_n_)/*2, refer to Figure 23, where *t_ep_* is the thickness of the end-plate, *t_cf_* is the thickness of the column web, *t_wh_* is the thickness of the bolt washer, and *t_h_* and *t_n_* are the thickness of the bolt head and nut, respectively.

#### 6.2.3. Integrated Connection Stiffness

Integrate the stiffness of the connection joint of the extended end-plate, according to the beam end rotation conforms to the plane-section assumption and the superposition principle of displacement in the elastic stage, the rotation angle *θ* can be expressed as:

(5) $$\theta =\theta_{epb}+\theta_{cfb}+\theta_{{}_{bt}}=\frac{M}{K_{con}}=\frac{M}{k_{epb}}+\frac{M}{k_{cfb}}+\frac{M}{k_{bt}}$$

According to the physical meaning of the initial rotational stiffness of the joint, the stiffness of the connection part of the extend end-plate is:

(6) $$k_{con}=\frac{1}{\frac{1}{k_{epb}}+\frac{1}{k_{cfb}}+\frac{1}{k_{bt}}}$$

In the formula, *M* is the elastic ultimate moments at the connection, *k_con_* is the connection stiffness, *k_epb_*, *k_cfb_*, and *k_bt_* are the bending stiffness of the end-plate, column web, and the tensile stiffness of the bolt, respectively.

### 6.3. Calculation of the Stiffness of the Column Web

#### 6.3.1. Tension and Compression Stiffness of the Column Web

According to the finite element analysis in the above section, it can be seen that the direction of force transmission, the axial force transmitted from the connection, causes the column web to be in a real state of compression and tension. The method of simplifying the column web to axial compression and axial tension plate can be used to calculate. Meanwhile, the influence of different load forms on the joint stiffness must also be considered. To calculate the initial rotational stiffness of the IC joints under the antisymmetric load and the EC joints under the asymmetric load.

**Figure 24:**
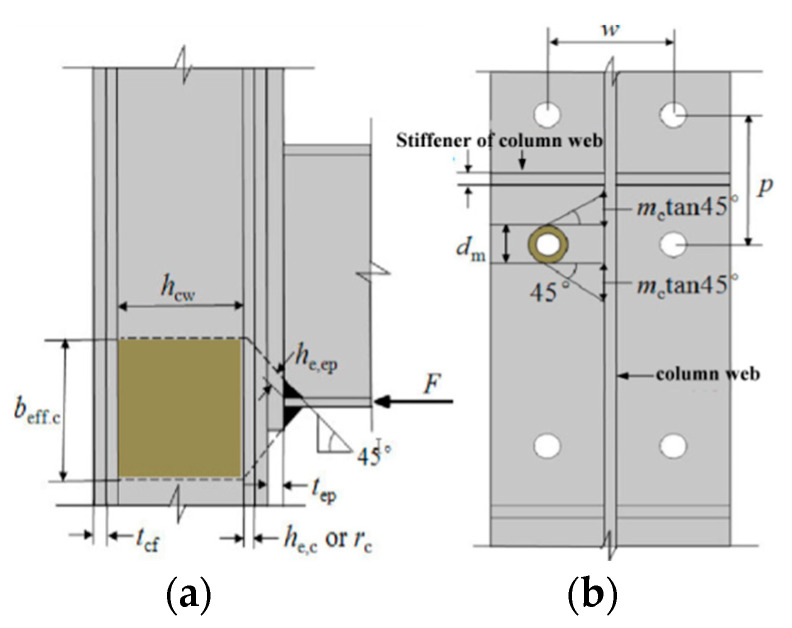
Effective width calculation of column web: (**a**) Effective compression width and (**b**) effective tension width.

The expression of the compression stiffness of the column web of the IC joint and EC joint is:

(7) $$k_{cwt}=\frac{Eb_{eff,t}t_{cw}}{\varphi h_{cw}}$$

where *h_cw_* is the calculated height of the column web (Figure 24a); *t_cw_* is the thickness of the column web; *b_eff,c_* is the effective compression width of the column web, reference [37], if the column is hot-rolled steel, then *b_eff,c_ = t_bf_ + 2h_e,ep_ + 2t_ep_ + 2(t_cf_ + r_c_)*; if the column is a welded steel section, *b_eff,c_ = t_bf_ + 2h_e,ep_ + 2t_ep_ + 2(t_cf_ + h_e,c_)*, where *h_e,ep_* and *h_e,c_* are the effective heights of the weld of the end-plate and column web, *r_c_* is the root radius of the column flange weld, *t_bf_* is the thickness of the beam flange, and the *φ* value is 0.5.

The formula for calculating the tensile stiffness of the IC joint and EC joint is shown in Equations (8) and (9).

(8) $$k_{cwt,\mathrm{IC}}=\lambda \frac{Eb_{eff,c}t_{cw}}{0.5\times h_{cw}}$$

(9) $$k_{cwt,\mathrm{EC}}=\lambda \frac{Eb_{eff,c}t_{cw}}{h_{cw}}$$

In the equation, IC and EC respectively represent the intermediate column joint and edge column joint; *λ* is to consider the influence coefficient of the bolt hole spacing, and the value *λ = (p/w)^3^*; and *b_eff,t_* is the effective tensile width of the column web. The calculation diagram is shown in Figure 24b. When *m_c_* is less than *(p* − *d_m_)/2*, then *b_eff,t_* is *2m_c_ + d_m_*; when *m_c_* is greater than or equal to *(p* − *d_m_)/2*, then *b_eff,t_* is *m_c_*tan45° *+ (d_m_ + p)/2*. Where *m_c_* is the distance from the center of the bolt hole to the welding foot of the column web, *p* and *w* are the vertical and horizontal distances between the centers of bolt holes, respectively. *dm* = *1.5* × *d_b_*, and *d_b_* is the nominal diameter of the bolt.

#### 6.3.2. Shear Stiffness of Column Web

**Figure 25:**
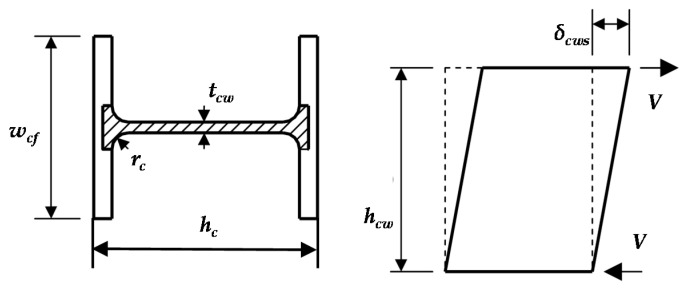
Shear area and shear deformation.

To calculate the deformation *δ_cws_* of the column web under shear, as shown in Figure 25, reference [38]. The column web can be assumed to be a short column that is only subjected to shear force *V*, which is generated by the moment of the connection at the upper and lower flanges of the beam. The deformation of the column web under the action of shear force is:

(10) $$\delta_{cws}=\frac{Vh_{cw}}{GA_{sc}}=2\times (1+\mu )\frac{V\beta h_{cw}}{EA_{sc}}$$

where *A_sc_* is the shear area of the column web, as shown in the shaded part in Figure 25. *A_sc_* = *h_c_h_cw_ −* *2w_cf_t_cf_* + *(t_cw_* + *2r_c_)t_cf_*, and *G* is the shear modulus of steel.

The shear stiffness of the web of the IC joint and EC joint is:

(11) $$k_{cws,IC}=\frac{1}{2\times (1+\mu )}\frac{EA_{sc}}{h_{cw}}$$

(12) $$k_{cws,EC}=\frac{1}{4\times (1+\mu )}\frac{EA_{sc}}{h_{cw}}$$

In the formula, when the connection belongs to the EC type, the column web is sheared on one side, and the β coefficient in the equation is 1; when the connection belongs to the IC joint type, the moments on both sides of the panel zone are equal and in the same direction, so the β coefficient in the equation is 2 if the moments on both sides of the panel zone are equal and in the opposite direction, so the β coefficient in the equation is 0, the bending moments offset each other, that is, no shear force. This paper does not involve such joints.

#### 6.3.3. Tension and Compression Stiffness of Column Web Stiffener

**Figure 26:**
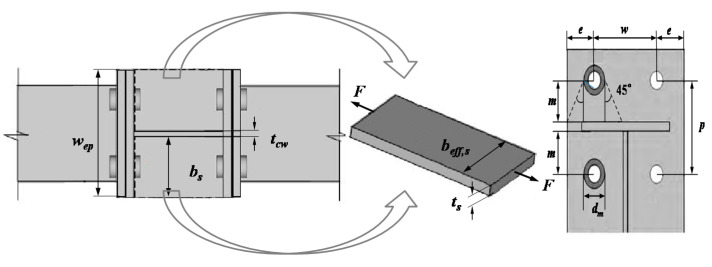
Calculation of effective width of stiffener of column web.

The stiffeners of the column webs also adopted the similar axial tension and compression method at the column webs to calculate the stiffness. As shown in Figure 26, according to the theory of material mechanics, the tensile and compressive stiffness is calculated.

Tensile stiffness of column web stiffener:

(13) $$k_{st}=\frac{Eb_{eff,st}t_{s}}{0.5\times h_{cw}}$$

Compression stiffness of column web stiffener:

(14) $$k_{sc}=\frac{2\times Eb_{eff,sc}t_{s}}{0.5\times h_{cw}}$$

where *t_s_* is the thickness of the stiffener of the column web; *b_eff,st_* and *b_eff,sc_* are the effective widths of the stiffener under tension and compression, respectively. Considering that the bolt force diffuses at 45° under tension.

Compression considers the pressure transmission in the direction of 45° along with the thickness of the end-plate and column flange at the lower flange of the beam. Effective width of stiffener under tension: *b_eff,st_* = *min{m_c_* + *e,d_m_* + *2m,e* + *m* + *d_m_/2,m_c_* + *m + d_m_/2,b_s_}*, where *m_c_* is the distance from the center of the bolt hole to the welding foot of the root of the column web, *e* is the horizontal distance from the center of the bolt hole to the external edge, *m* is the distance from the bolt hole to the surface of the beam flange, *dm* = *1.5* × *d_b_*, *d_b_* is the nominal diameter of the bolt; effective width of stiffener under compression *b_eff,sc_*: if *(w_ep_* – *w_bf_)/2 ≥ t_ep_*, then *b_eff,sc_* = *min{w_bf_/2* + *t_cf_* + *t_ep_,w_bf_/2}*, if *(w_ep_* – *w_bf_)/2*<*t_ep_*, then *b_eff,sc_* = *min{w_ep_/2* + *t_cf_,b_cf_/2}*. *w_ep_*, *w_bf_*, and *w_cf_* are the width of the connecting end-plate, the width of the beam flange, and the width of the column flange, respectively; *b_s_* is the width of the stiffener and *t_ep_* is the thickness of the end-plate. The above specific parameters are shown in Figure 26.

#### 6.3.4. Integrated Column Web Stiffness

The shear, tension, and compression deformation of the column web and the tension and compression deformation of the stiffeners cause relative deformation between the panel zones, which in turn causes the beam ends to rotate. Under the action of the moment *M*, the five deformations of the column web are:

(15) $$\delta_{cws}=\frac{M}{h_{0}K_{cws}};\delta_{cwt}=\frac{M}{h_{0}k_{cwt}};\delta_{cwc}=\frac{M}{h_{0}k_{cwc}};\delta_{st}=\frac{M}{h_{0}k_{st}};\delta_{sc}=\frac{M}{h_{0}k_{sc}}$$

The rotation produced by the column web is:

(16) $$\theta_{cw}=\left(\delta_{cwc}+\delta_{cwt}+\delta_{cwv}+\delta_{st}+\delta_{sc}\right)/h_{0}$$

The overall stiffness of the column web is:

(17) $$K_{cw}=\frac{h_{0}^{2}}{\frac{1}{k_{cwc}}+\frac{1}{k_{cwt}}+\frac{1}{k_{cwv}}+\frac{1}{k_{sc}}+\frac{1}{k_{st}}}$$

In the formula, *h*_0_ is the vertical distance between the center of the upper and lower flanges of the beam, *h*_0_ = *h_b_* – *t_bf_*, *h_b_* is the height of the beam, and *t_bf_* is the thickness of the beam flange.

### 6.4. Validation

First applied to the test specimens IC-EP1/2/3 and EC-EP1/2/3, to verify the improved component model for predicting the initial rotational stiffness, Table 7 summarizes the results of the component method model, tests, and the corresponding FE model. Compared with the experimental and finite element results, the theoretical predictions are consistent, and the error margin for individual specimens is 16%.

To further validate the above-mentioned component model. The stiffness of the 144 finite element models of the above parameter analysis has been calculated. The model included parameters such as different column section sizes, end-plate thickness, bolt diameter, and end-plate size to verify the proposed component method. Table 8 compares the stiffness of FE analysis and component method calculations. Based on the comparative analysis of 144 FE models and component method calculations, *Δ* represents the ratio of the difference between the two to FE, that is *Δ* = *|K_T_ − K_FE_|/K_FE_*. For the average of this ratio, the relative difference between the two is 8.99%. For dispersion, the standard deviation of this relative difference is equal to 6.88%. Due to the complexity and high cost of the test, the value calculated by the component method is still acceptable. The above two groups of verification data verify the validity of the initial rotational stiffness’s theoretical calculation. So generally, the theoretical equation proposed in this article is of good applicability for most reasonably designed joints.

## 7. Conclusions

This study involved analyzing the performance difference between IC joints and EC joints of extended end-plate connection under the column top-side loading method. Firstly, based on the test data of six joints, a set of symmetrical models was developed to verify actual experimental results, and the failure modes and joint stiffness were analyzed and compared. The above work laid the foundation for parameter analysis; a total of 144 symmetrical 3D finite element models were created to study the influence of cyclic loads on the extended end plate beam-column connection with different parameters. Finally, a calculation equation for the initial rotation stiffness of the joints was presented based on the component method. The key conclusions of the study include the following:

- According to the influence of different parameters on the mechanical behavior of the extended end-plate connection, the parametric FE models were established, and the effects on the flexural capacity, rotational stiffness and limit rotation of the joints are studied. Meanwhile, the joint typical failure model is divided into six types. It is also found that the joints also show nonlinear characteristics in the early stage, and some connected components have already yielded in the initial stage, and each component will not respond uniformly as a whole. The behavior of joints in the whole process is nonlinear.
- In most studies in the past, the main focus has been on the study of the moment-rotation relationship of the connection under the beam tip loading method, but this article focuses on some force forms in actual engineering, using the column top-side loading method to study the parameter’s comprehensive impact on joint performance. Additionally, the research on the panel zone shearing effect is focused, and it is concluded that the value of shearing force acting on the connection has a great influence on the mechanical behaviors of joints in this type of loading. Meanwhile, the shear rotation of the IC joint parameter model accounts for about 2/3 of the total connection rotation times, and the shear force in the panel zone is twice that of the EC joint. However, under the control of the failure mode of the weakest component, the ultimate flexural resistance capacity of the IC joint and EC joint remains consistent.
- Increasing the diameter of bolts or the thickness of the end-plate in most cases enhances the behavior of the connection, in other words, increases both moment capacity and rotation capacity by a certain percentage. However, the yield rotation of the connection is decreased with an increase in the thickness of end-plates, resulting in low connection ductility. In spite of this, the use of medium-thickness end-plates with bolts of appropriate diameters can greatly improve the overall mechanical properties of joints.
- According to the mechanical behavior of the joint components. The component model of the joint under the column top-side loading method is proposed, and the initial stiffness expression is established. The bending stiffness of the component was improved by adopting the large deflection calculation of the plate and shell theory, and considering the significant difference in the contribution of the shear stiffness component of the panel zone between the IC joint and EC joint. The expression is verified by experimental results and the 144-parameter FE model, which proves the reliability of the initial stiffness expression.

## Figures and Tables

**Table 1:** Configuration details of joint specimens.

Joints Type	Specimen	End-Plate Thickness (mm)	Bolt Diameter (mm)	Beam Section (mm)	Column Section (mm)	*F_pre_* (kN)	*F_c_* (kN)
IC joint	IC-EP1	16	20	H300 × 200 × 8 × 12	H300 × 300 × 10 × 15	155	1250
	IC-EP2	16	24	H300 × 200 × 8 × 12	H300 × 300 × 10 × 15	225	1250
	IC-EP3	20	20	H300 × 200 × 8 × 12	H300 × 300 × 10 × 15	155	1250
EC joint	EC-EP1	16	20	H300 × 200 × 8 × 12	H300 × 300 × 10 × 15	155	1250
	EC-EP2	20	20	H300 × 200 × 8 × 12	H300 × 300 × 10 × 15	155	1250
	EC-EP3	12	20	H300 × 200 × 8 × 12	H300 × 300 × 10 × 15	155	1250

Table note: IC joint and EC joint represent the intermediate column joint and edge column joint respectively.

**Table 2:** Main test results.

Specimen		*K_ji_* (kN·m/mrad)	*M_y_* (kN·m)	*M_max_* (kN·m)	*θ_y_* (mrad)	*θ_u_* (rad)	*μ_θ_*	Failure Pattern
IC-EP1	west	55.8	130.4	251.1	6.7	0.06	9.0	The weld cracked at the flange and the bolt broke.
	east	57.5	124.1	239.1	6.5	0.06	9.2	
IC-EP2	west	63.3	138.6	239.8	7.9	0.07	8.9	The large shear deformation of panel zone.
	east	62.6	112.3	211.7	7.1	0.06	8.5	
IC-EP3	west	43.3	128.5	209.8	7.3	0.07	9.6	Moderate shear deformation of panel zone and bolt breakage.
	east	38.6	132.1	228.7	6.9	0.07	10.1	
EC-EP1	/	25.7	130.7	236.6	4.7	0.05	10.6	The excessive end-plate bending.
EC-EP2	/	27.6	120.1	225.8	5.1	0.05	9.8	
EC-EP3	/	21.7	110.1	207.4	5.4	0.06	11.1	

Table note: *K_ji_* is the initial rotational stiffness of the joints; *M_y_, M_max_, θ_y_, and θ_u_* are defined by the key parameters of the connections; *μ_θ_* is the ductility coefficient of the test specimen; and *μ_θ_* = *θ_u_*/*θ_y_*.

**Table 3:** Bolt and steel material properties [31].

Material Type	*f_y_*/MPa	*f_u_*/MPa	*ε_y_*/%	E/GPa	E_st_/GPa
Q345B steel	370.16	556.20	0.40	207.27	0.02E
10.9-grade high strength bolt	987.55	1182.10	0.50	208.23	0.11E

**Table 4:** Comparison of finite element (FE) symmetry models of IC and EC.

Joints Type	FE Model	Symmetry	Corresponding Figure
IC joint	FE all, FE 1/2 and FE 1/4	Boundary symmetry Load antisymmetric	Figure 6a–c
EC joint	FE all and FE 1/2	Boundary symmetry Load asymmetry	Figure 7a,b

**Table 5:** Comparison of the FE analysis and test results.

Specimen	Method	*K_ji_* (kN·m/mrad)	*M_y_* (kN·m)	*θ_y_* (mrad)	*K_FE_/K_Test_*	Method	*K_ji_* (kN·m/mrad)	*M_y_* (kN·m)	*θ_y_* (mrad)	*K_FE_/K_Test_*
IC-EP1/west	Test	55.8	130.4	6.7	/	FE 1/2	53.5	123.2	5.3	0.96
	FE all	57.8	136.7	6.1	1.04	FE 1/4	52.2	155.8	5.7	0.94
IC-EP2/west	Test	63.3	138.6	7.9	/	FE 1/2	65.6	150.6	6.7	1.04
	FE all	64.9	146.3	7.8	1.03	FE 1/4	66.1	163.2	5.5	1.04
IC-EP3/west	Test	43.3	128.5	7.3	/	FE 1/2	42.7	130.8	7.1	0.99
	FE all	45.6	137.6	6.9	1.05	FE 1/4	47.3	135.4	6.8	1.09
EC-EP1	Test	25.7	130.7	4.7	/	FE 1/2	28.5	153.8	4.1	1.11
	FE all	26.3	141.3	5.3	1.02					
PEC-EP2	Test	27.6	120.1	5.1	/	FE 1/2	29.8	135.7	3.7	1.08
	FE all	30.9	130.5	4.9	1.12					
EC-EP3	Test	21.7	110.1	5.4	/	FE 1/2	22.7	117.4	5.6	1.05
	FE all	23.4	105.6	4.7	1.08					

**Table 6:** Parametric analysis.

Num	Model Label	*K_ji_*	*M_y_*	*M_max_*	*θ_y_*	*θ_u_*	Failure Mode	Num	Model Label	*K_ji_*	*M_y_*	*M_max_*	*θ_y_*	*θ_u_*	Failure Mode
		(kN·m/mrad)	(kN*·*m)		(mrad)					(kN·m/mrad)	(kN*·*m)		(mrad)		
1	ICBC1D16t12L8S1	47.7	103	164.6	3.4	81	EPF	37	ICBC2D16t12L8S1	25.9	76	106.6	4.3	68.2	EPF
2	ICBC1D16t12L8S2	52.4	169.2	237.7	3.5	69.9	EPF	38	ICBC2D16t12L8S2	36.8	125.8	180.1	7.5	68.8	EPF
3	ICBC1D16t12L15S1	52.7	112.4	183.4	7.2	79	EPF	39	ICBC2D16t12L15S1	25	83	115.4	7.6	71.3	EPF
4	ICBC1D16t12L15S2	61.9	178.4	264.8	2.8	70.7	EPF	40	ICBC2D16t12L15S2	40.9	142.4	205.5	6.8	67	EPF
5	ICBC1D16t16L8S1	63.1	179.4	258.5	6	77	CPZB	41	ICBC2D16t16L8S1	44.1	139	222.5	7	59	WEP-BF
6	ICBC1D16t16L8S2	69.6	182.4	278.5	5.3	72.4	CFF	42	ICBC2D16t16L8S2	43.9	146.4	223.5	8.8	55.4	CFF
7	ICBC1D16t16L15S1	71.2	182.6	234.3	3.4	75.4	CPZB	43	ICBC2D16t16L15S1	47.8	146.6	194.2	3.8	54.5	CFF
8	ICBC1D16t16L15S2	71.7	179.2	257.2	2.9	72.6	CFF	44	ICBC2D16t16L15S2	45.1	137.7	207.9	3.4	56.7	CFF
9	ICBC1D16t20L8S1	87.5	182.8	240.3	5.4	68.5	BF	45	ICBC2D16t20L8S1	36.5	147.4	222.3	5.4	66.9	BF
10	ICBC1D16t20L8S2	90.1	189.7	286.2	6.3	67.8	BF	46	ICBC2D16t20L8S2	55.6	151.2	210.8	7.1	61.4	BF
11	ICBC1D16t20L15S1	91.2	195.6	300.7	3	77.7	BF	47	ICBC2D16t20L15S1	54.3	147.5	222	5.2	59.3	BF
12	ICBC1D16t20L15S2	94.6	199.4	264.3	4.7	71	BF	48	ICBC2D16t20L15S2	57	155.2	226.7	8.3	50.4	BF
13	ICBC1D20t12L8S1	49.3	147.3	208.8	3.6	78.3	EPF	49	ICBC2D20t12L8S1	21	116.5	171.2	6.8	68.2	EPF
14	ICBC1D20t12L8S2	53.5	169.4	255.8	6.6	76.8	EPF	50	ICBC2D20t12L8S2	30.6	129.1	170.9	6	70.4	EPF
15	ICBC1D20t12L15S1	45.6	157.4	274.3	6.1	62.5	EPF	51	ICBC2D20t12L15S1	22.3	127.4	199.4	9.3	78	EPF
16	ICBC1D20t12L15S2	56	180.9	265.2	5.3	68.8	EPF	52	ICBC2D20t12L15S2	28.5	140.4	218.7	3.8	72.4	EPF
17	ICBC1D20t16L8S1	57.8	179.4	218.5	7.2	71.9	WCF-CS	53	ICBC2D20t16L8S1	38.7	146.7	163.7	4.8	60.4	CFF
18	ICBC1D20t16L8S2	62.7	189.7	276.5	5.8	56.2	WEP-BF	54	ICBC2D20t16L8S2	39.3	150.1	206.6	6.7	55.5	WEP-BF
19	ICBC1D20t16L15S1	52.2	165.6	240.1	4.6	53.7	CFF	55	ICBC2D20t16L15S1	26.4	125.6	215.1	7	69.5	CFF
20	ICBC1D20t16L15S2	57.6	181.8	270.6	3.4	65.4	EPF	56	ICBC2D20t16L15S2	42.1	142.6	248.1	4.8	63.6	CPZB
21	ICBC1D20t20L8S1	63.2	187.8	285.7	8.3	65.1	CFF	57	ICBC2D20t20L8S1	35.3	144.1	201.3	7.6	56.8	WCF-CS
22	ICBC1D20t20L8S2	65.1	189.7	253	7.9	63	WEP-BF	58	ICBC2D20t20L8S2	32.1	142.1	212.2	5	54.8	WEP-BF
23	ICBC1D20t20L15S1	61.8	199.4	290.6	7.7	53	CPZB	59	ICBC2D20t20L15S1	41	153.2	259.4	8.2	51.6	CPZB
24	ICBC1D20t20L15S2	65.9	211.2	295.5	8.8	56.9	CPZB	60	ICBC2D20t20L15S2	36.4	163.9	207.1	4	65.5	CPZB
25	ICBC1D24t12L8S1	39.1	155.9	202.4	7.4	73.9	EPF	61	ICBC2D24t12L8S1	18.8	125.1	198.7	4.7	60.7	EPF
26	ICBC1D24t12L8S2	41.9	168.9	257.9	9	75.1	EPF	62	ICBC2D24t12L8S2	30.3	130	187.4	3.2	57.8	EPF
27	ICBC1D24t12L15S1	45.2	163.8	247.4	7.1	78.9	EPF	63	ICBC2D24t12L15S1	25.2	122.3	215.1	7.5	72.7	EPF
28	ICBC1D24t12L15S2	46.8	178.2	258.3	4	77	EPF	64	ICBC2D24t12L15S2	30.9	135.1	205.3	9.1	70.3	EPF
29	ICBC1D24t16L8S1	60.2	178.8	253.9	5.6	62.7	WCF-CS	65	ICBC2D24t16L8S1	31	137.5	191.9	2.9	59.8	WEP-BF
30	ICBC1D24t16L8S2	62.1	201	289.1	5.2	61.2	WEP-BF	66	ICBC2D24t16L8S2	34.1	162.3	204	4.4	58.4	WCF-CS
31	ICBC1D24t16L15S1	66.1	165.8	309.6	6.1	60	EPF	67	ICBC2D24t16L15S1	31.5	130.9	230.8	5.9	56.4	WEP-BF
32	ICBC1D24t16L15S2	45.6	192.7	248.6	3.8	53.3	CFF	68	ICBC2D24t16L15S2	35.1	148.2	224.2	3.1	53.6	CPZB
33	ICBC1D24t20L8S1	72.2	189.1	285.9	6.1	67.1	WCF-CS	69	ICBC2D24t20L8S1	51.2	152.3	234.6	3.3	59.9	WCF-CS
34	ICBC1D24t20L8S2	71.2	203.1	284.9	4.1	64.3	WEP-BF	70	ICBC2D24t20L8S2	44	156.8	231.2	8.4	55.5	CFF
35	ICBC1D24t20L15S1	74.4	207.6	297.2	7.3	63.7	CPZB	71	ICBC2D24t20L15S1	46.2	163.2	229.7	8	54.6	CPZB
36	ICBC1D24t20L15S2	76.5	218.6	295.7	5.5	62.4	CPZB	72	ICBC2D24t20L15S2	47.1	168.3	218.2	6.9	52.6	CPZB
73	ECBC1D16t12L8S1	22.3	99.3	154	6.4	62.9	EPF	109	ECBC2D16t12L8S1	22.5	66.9	112.3	8.3	54.9	EPF
74	ECBC1D16t12L8S2	30.6	154.2	168.4	6.7	54.2	EPF	110	ECBC2D16t12L8S2	31.4	115.4	163.4	6.1	52.4	EPF
75	ECBC1D16t12L15S1	30.7	111.9	179.3	4.4	62.7	EPF	111	ECBC2D16t12L15S1	21.2	71.4	126.5	5.9	55.7	EPF
76	ECBC1D16t12L15S2	34.5	169.4	171.2	3.6	53.3	EPF	112	ECBC2D16t12L15S2	27.3	140.7	214.6	6	50.7	EPF
77	ECBC1D16t16L8S1	31.2	168.6	182.5	7.2	60.1	WCF-CS	113	ECBC2D16t16L8S1	27.8	129	196.2	3.3	46.6	EPF
78	ECBC1D16t16L8S2	31.5	176.6	189.7	9	53	WEP-BF	114	ECBC2D16t16L8S2	32.7	135.6	225.3	3.5	46.1	WEP-BF
79	ECBC1D16t16L15S1	31.5	182.1	216.6	4.1	59	CFF	115	ECBC2D16t16L15S1	23.8	141.8	216.1	7.4	42.2	CFF
80	ECBC1D16t16L15S2	34.1	172.1	210.4	7.8	56.7	CFF	116	ECBC2D16t16L15S2	28.5	134.3	187.4	3.2	45.4	CPZB
81	ECBC1D16t20L8S1	36.9	174.2	216	5	55	BF	117	ECBC2D16t20L8S1	28.2	148.2	203.8	6.4	49.5	BF
82	ECBC1D16t20L8S2	43.3	180.5	215	6.5	50.5	BF	118	ECBC2D16t20L8S2	29.2	145.4	213.2	6.9	51.1	BF
83	ECBC1D16t20L15S1	41.9	190.3	224.6	8.6	61.6	BF	119	ECBC2D16t20L15S1	29.8	142.8	220	5.7	45.9	BF
84	ECBC1D16t20L15S2	45	195.6	225.2	8.7	55	BF	120	ECBC2D16t20L15S2	33.6	141.5	223.7	8.3	40.3	BF
85	ECBC1D20t12L8S1	27.1	145	228	4.2	59.9	EPF	121	ECBC2D20t12L8S1	20.2	110.7	165.5	4.2	54.8	EPF
86	ECBC1D20t12L8S2	24.4	171.7	230.9	8.4	55.5	EPF	122	ECBC2D20t12L8S2	23.8	118.5	162.7	6.3	50.8	EPF
87	ECBC1D20t12L15S1	28	164.6	225.8	8.9	47.3	EPF	123	ECBC2D20t12L15S1	19.9	142.5	161.8	8.5	60.1	EPF
88	ECBC1D20t12L15S2	28.9	171.8	275.5	8.4	55.5	EPF	124	ECBC2D20t12L15S2	26.4	129.5	208.8	5.1	59.7	EPF
89	ECBC1D20t16L8S1	38.9	181.2	283.7	6.7	56.8	WEP-BF	125	ECBC2D20t16L8S1	22.1	133.3	262.3	2.7	45.2	WEP-BF
90	ECBC1D20t16L8S2	41.9	186.6	317.7	7.1	41.9	WEP-BF	126	ECBC2D20t16L8S2	27.6	135.4	278.9	3.2	39.9	WEP-BF
91	ECBC1D20t16L15S1	28.5	165.7	233.4	2.7	44	EPF	127	ECBC2D20t16L15S1	27.4	122.1	197.1	8.1	50.6	EPF
92	ECBC1D20t16L15S2	31.2	171.6	305.2	7.3	47.2	EPF	128	ECBC2D20t16L15S2	31.6	134.7	208.1	7.1	51.5	CFF
93	ECBC1D20t20L8S1	45	181.6	230	6.7	47.5	WEP-BF	129	ECBC2D20t20L8S1	26.7	143.9	203.4	4.9	48.1	EPF
94	ECBC1D20t20L8S2	43.6	182.3	235.7	4.6	47	WCF-CS	130	ECBC2D20t20L8S2	29.4	139.3	183.8	3.2	42.2	WCF-CS
95	ECBC1D20t20L15S1	29.8	180.4	274.8	3.1	43.5	CFF	131	ECBC2D20t20L15S1	29.6	151.6	201.8	6.1	41.5	CPZB
96	ECBC1D20t20L15S2	33.9	200	233.1	3.6	46.5	CPZB	132	ECBC2D20t20L15S2	32.3	161.5	182.5	7.1	50.4	WCF-CS
97	ECBC1D24t12L8S1	22.3	141.3	229.4	4.8	55.6	EPF	133	ECBC2D24t12L8S1	21.1	121.5	175.9	5	50	EPF
98	ECBC1D24t12L8S2	21.4	164.4	245.3	6.3	60	EPF	134	ECBC2D24t12L8S2	27.3	126.5	179.4	7.1	42.3	EPF
99	ECBC1D24t12L15S1	22.9	162.3	223.2	9	59.4	EPF	135	ECBC2D24t12L15S1	30.2	113	196.1	5.9	53.9	EPF
100	ECBC1D24t12L15S2	29.6	176.5	251.6	5	58.9	EPF	136	ECBC2D24t12L15S2	32.5	127.6	220	8.4	53.9	EPF
101	ECBC1D24t16L8S1	36.2	165.2	263.2	7.6	47.2	EPF	137	ECBC2D24t16L8S1	22.5	129.9	214.4	2.7	45.1	EPF
102	ECBC1D24t16L8S2	39.3	193.7	302.7	3.6	47.3	WEP-BF	138	ECBC2D24t16L8S2	28.7	148.3	239.6	7.1	49.5	WEP-BF
103	ECBC1D24t16L15S1	36.8	161.5	271.1	7.4	45	CPZB	139	ECBC2D24t16L15S1	32.9	127.7	209.6	7.1	45.7	EPF
104	ECBC1D24t16L15S2	48.1	182	288.3	7.2	40.7	CFF	140	ECBC2D24t16L15S2	34.8	140.5	202.8	3.6	43.7	WCF-CS
105	ECBC1D24t20L8S1	48.2	187	300.2	3.7	51	WEP-BF	141	ECBC2D24t20L8S1	30.1	148	210.2	3.4	47.8	CFF
106	ECBC1D24t20L8S2	51.1	195.4	289.8	6.6	49	WCF-CS	142	ECBC2D24t20L8S2	33.6	148.1	213.9	8.9	40.8	CFF
107	ECBC1D24t20L15S1	55.2	202	276.9	4.2	47.2	CPZB	143	ECBC2D24t20L15S1	31.3	159.7	268.8	6.5	44	CPZB
108	ECBC1D24t20L15S2	56.2	209.8	306.5	4.6	47.3	WEP-BF	144	ECBC2D24t20L15S2	32.9	156.6	267.4	9	39.3	WCF-CS

Table note: Initial rotational stiffness, yield moment, maximum moment, yield rotation, ultimate rotation, and failure mode for the 144 FE models used in the parametric study. EPF, CPZB, WCF-CS, etc. represent joints failure modes. Refer to Section 5.2.1 for specific meanings.

**Table 7:** Comparison between the component method and FE analysis results for connection initial rotational stiffness.

Specimen	Component Model-*K_theory_*	Test-*K_Test_*	FE-*K_FE(all)_*	Ratio *K_theory_*/*K_Test_*	Ratio *K_theory_*/*K_FE(all)_*
	(kN·m/mrad)				
IC-EP1/west	51.5	55.8	57.8	0.92	0.99
IC-EP2/west	68.9	63.3	64.9	1.09	0.92
IC-EP3/west	47.1	43.3	44.9	1.09	1.05
EC-EP1	26.3	25.7	26.3	1.02	0.94
EC-EP2	31.9	27.6	30.8	1.16	1.04
EC-EP3	23.4	21.7	22.8	1.08	1.03

**Table 8:** Initial rotational stiffness finite element and theoretical calculation results.

Model Num	*K_ji_* (kN·m/mrad)		*Δ* (%)	Model Num	*K_ji_* (kN·m/mrad)		*Δ* (%)	Model Num	*K_ji_* (kN·m/mrad)		*Δ* (%)	Model Num	*K_ji_* (kN·m/mrad)		*Δ* (%)	Model Num	*K_ji_* (kN·m/mrad)		*Δ* (%)	Model Num	*K_ji_* (kN·m/mrad)		*Δ* (%)
	FE	Theory			FE	Theory			FE	Theory			FE	Theory			FE	Theory			FE	Theory	
001	47.7	51.2	7.3	025	39.1	38.9	0.5	049	21.0	22.0	4.8	073	22.3	26.2	17.5	097	22.3	26.4	18.4	121	20.2	20.4	1.0
002	52.4	55.5	5.9	026	41.9	44.1	5.3	050	30.6	31.6	3.3	074	30.6	35.3	15.4	098	21.4	25.1	17.3	122	23.8	25.0	5.0
003	52.7	51.2	2.8	027	45.2	38.9	13.9	051	22.3	22.0	1.3	075	30.7	26.2	14.7	099	22.9	26.4	15.3	123	19.9	20.4	2.5
004	61.9	55.5	10.3	028	46.8	44.1	5.8	052	28.5	31.6	10.9	076	34.5	35.3	2.3	100	29.6	25.1	15.2	124	26.4	25.0	5.3
005	63.1	66.9	6.0	029	60.2	68.9	14.5	053	38.7	31.7	18.1	077	31.2	31.7	1.6	101	36.2	34.0	6.1	125	22.1	23.5	6.3
006	69.6	70.6	1.4	030	62.1	66.3	6.8	054	39.3	37.1	5.6	078	31.5	36.8	16.8	102	39.3	44.1	12.2	126	27.6	31.0	12.3
007	71.2	66.9	6.0	031	66.1	68.9	4.2	055	26.4	31.7	20.1	079	31.5	31.7	0.6	103	36.8	34.0	7.6	127	27.4	23.5	14.2
008	71.7	70.6	1.5	032	45.6	46.3	1.5	056	42.1	37.1	11.9	080	34.1	36.8	7.9	104	48.1	44.1	8.3	128	31.6	31.0	1.9
009	87.5	93.9	7.3	033	72.2	78.9	9.3	057	35.3	37.4	5.9	081	36.9	34.4	6.8	105	48.2	48.7	1.0	129	26.7	26.9	0.7
010	90.1	98.0	8.8	034	71.2	77.2	8.4	058	32.1	35.6	10.9	082	43.3	37.4	13.6	106	51.1	49.4	3.3	130	29.4	31.4	6.8
011	91.2	93.9	3.0	035	74.4	78.9	6.0	059	41.0	37.4	8.8	083	41.9	34.4	17.9	107	55.2	48.7	11.8	131	29.6	26.9	9.1
012	94.6	98.0	3.6	036	76.5	77.2	0.9	060	36.4	35.6	2.2	084	45.0	37.4	16.9	108	56.2	49.4	12.1	132	32.3	31.4	2.8
013	49.3	51.5	4.5	037	25.9	22.0	15.1	061	18.8	22.1	17.6	085	27.1	26.3	3.0	109	22.5	20.3	9.8	133	21.1	24.4	15.6
014	53.5	65.9	23.2	038	36.8	35.3	4.1	062	30.3	31.6	4.3	086	24.4	26.0	6.6	110	31.4	29.5	6.1	134	27.3	29.9	9.5
015	45.6	51.5	12.9	039	25.0	22.0	12.0	063	25.2	22.1	12.3	087	28.0	26.3	6.1	111	21.2	20.3	4.2	135	30.2	24.4	19.2
012	56.0	65.9	17.7	040	40.9	35.3	13.7	064	30.9	31.6	2.3	088	28.9	26.0	10.0	112	27.3	29.5	8.1	136	32.5	29.9	8.0
017	57.8	77.7	34.4	041	44.1	45.6	3.4	065	31.0	28.8	7.1	089	38.9	31.9	18.0	113	27.8	23.4	15.8	137	22.5	23.5	4.4
018	62.7	66.9	6.7	042	43.9	46.7	6.4	066	34.1	37.1	8.8	090	41.9	37.5	10.5	114	32.7	30.5	6.7	138	28.7	31.0	8.0
019	52.2	51.5	1.3	043	47.8	45.6	4.6	067	31.5	28.8	8.6	091	28.5	26.3	7.7	115	23.8	23.4	1.7	139	32.9	23.5	28.6
020	57.6	66.9	16.1	044	45.1	46.7	3.5	068	35.1	37.1	5.7	092	31.2	37.5	20.2	112	28.5	30.5	7.0	140	34.8	31.0	10.9
021	63.2	51.5	18.5	045	36.5	45.3	24.1	069	51.2	47.4	7.4	093	45.0	41.6	7.6	117	28.2	27.8	1.4	141	30.1	28.9	4.0
022	65.1	66.7	2.5	046	55.6	57.2	2.9	070	44.0	47.6	8.2	094	43.6	38.1	12.6	118	29.2	30.9	5.8	142	33.6	31.4	6.5
023	61.8	51.5	16.7	047	54.3	45.3	16.6	071	46.2	47.4	2.6	095	29.8	31.9	7.0	119	29.8	27.8	6.7	143	31.3	28.9	7.7
024	65.9	66.7	1.2	048	57.0	57.2	0.4	072	47.1	47.6	1.1	096	33.9	38.1	12.4	120	33.6	30.9	8.0	144	32.9	31.4	4.6

Table note: *Δ* = |*K_T_* − *K_FE_*|/*K_FE_*, *K_T_* and *K_FE_* represent initial rotational stiffness of the FE and the Theory, the standard deviation and mean of *Δ* are 8.99% and 6.88%, respectively.

## Nomenclature

**Symbols**	**Content**	**Symbols**	**Content**
*a:*	The calculated length of the cantilever rectangular plate	*a_cf_:*	The calculated length of the column flange T-stub
*a_ep_:*	The calculated length of the end-plate T-stub	*A_s_:*	The effective area of the bolt rod
*A_sc_:*	The shear area of the column web	*b:*	The calculated height of the cantilever rectangular plate
*b_cf_:*	Calculate the height of column flange T-stub	*b_eff,c_:*	Effective height of the column web in compression
*b_eff,sc_:*	The effective compression width of column web stiffener	*b_eff,st_:*	The effective tensile width of column web stiffener
*b_eff,t_:*	The effective height of the column web in tension	*b_ep_:*	The calculated height of the end-plate T-stub
*b_s_:*	The width of the column web stiffener	*D:*	The unit width of the plate bending rigidity
*d_b_:*	The nominal diameter of the bolt	*d_m_:*	The effective diameter of the bolt head, *d_m_* = 1.5*d_b_*
*e:*	The horizontal distance from the center of the bolt hole to the external edge	*E:*	Elasticity modulus
*E_st_:*	Elastoplastic modulus	*h_b_:*	The height of beam section
*h_c_:*	The height of the column section	*h_cw_:*	Calculation height of the column web
*h_cw,c_:*	The equivalent calculation height of the column web in compression	*h_cw,t_:*	The equivalent calculation height of column web in tension
*h_e,c_:*	Effective heights of welded of the column	*h_e,ep_:*	Effective heights of welded of the end-plate
*k_bt_:*	The tensile stiffness in the height of each row of bolts	*k_cfb_:*	The bending stiffness of the column flange
*K_con_:*	The stiffness of the connection part	*K_cw_:*	The stiffness of the column web part
*k_cwc_:*	The compressive stiffness of column web	*k_cws_:*	The shear stiffness of column web
*k_cwt_:*	The tensile stiffness of column web	*k_epb_:*	The bending stiffness of end-plate
*K_ji_:*	Joint initial rotation stiffness	*k_sc_:*	The compressive stiffness of column web stiffener
*k_st_:*	The tensile stiffness of column web stiffener	*L_b_:*	The calculation length of the bolt
*l_eff,cf_:*	The effective lengths of the equivalent T-stubs of the column flange	*l_eff,ep_:*	The effective lengths of the equivalent T-stubs of the end-plate
*m:*	The distance from the bolt hole to the surface of the beam flange	*M:*	Elastic ultimate moments
*m_c_:*	The distance from the center of the bolt hole to the welding foot of the column web	*p:*	Horizontal distance bolt holes
*r_c_:*	The root radius of the column flange weld	*t_bf_:*	The thickness of the beam flange
*t_cf_:*	The thickness of the column flange	*t_cw_:*	The thickness of the web
*t_ep_:*	The thickness of the end-plate	*t_h_:*	The thickness of bolt head
*t_n_:*	The thickness of the bolt nut	*t_s_:*	The thickness of column web stiffener
**Symbols**	**Content**	**Symbols**	**Content**
*t_wh_:*	The thickness of the bolt washer	*w:*	The vertical distance between bolt holes
*w_bf_:*	Width of the beam flange	*w_cf_:*	Width of the column flange
*w_ep_:*	Width of the end-plate	*α:*	The rectangular plate coefficient related to the length and constraints
*γ:*	The coefficient of prestress effect	*θ_u_:*	Ultimate rotation
*λ:*	The influence coefficient of the bolt hole spacing, and the value *λ* = (*p*/*w*)^3^	*μ:*	The Poisson’s ratio of the steel
*Φ:*	The influence coefficient of the joints type	*ω_epb1_:*	The center deflection of the No.I plate of end-plate T-stub
*ω_epb2_:*	The center deflection of the No. II plate of end-plate T-stub	*ω_m_:*	The center deflection of the cantilever rectangular plate
*△_b_:*	The bending deformation of the end-plate and column flange	*△_c_:*	The compressive deformation of the column web
*△_s_:*	The shear deformation of the column web	*△_t_:*	The tensile deformation of the bolts
